# CAST/ELKS–endophilin-A interaction ensures synaptic vesicle pool size

**DOI:** 10.1083/jcb.202508077

**Published:** 2026-07-22

**Authors:** Yasunori Mori, Hirokazu Sakamoto, Yeon-Jeong Kim, Shun Hamada, Fumihiro Kaku, Yamato Hida, Kenzo Hirose, Toshihisa Ohtsuka

**Affiliations:** 1 Department of Biochemistry, Graduate School of Medicine/Faculty of Medicine, University of Yamanashi, Chuo, Japan; 2 Department of Pharmacology, Graduate School of Medicine, The University of Tokyo, Tokyo, Japan; 3 Department of Cell Physiology, Graduate School of Medicine, Nagoya University, Nagoya, Japan

## Abstract

Efficient neurotransmission relies on the precise integration of synaptic vesicle (SV) recycling at the presynaptic terminal. While SV recycling is essential for neurotransmission, the molecular mechanisms coordinating vesicle dynamics at presynaptic terminals remain poorly understood. Here we report that the active zone proteins, CAST and ELKS, play a crucial role in maintaining functional SV pool size through direct interaction with endophilin-A family proteins. In cultured hippocampal neurons, disruption of CAST binding to endophilin-A resulted in a reduced number of SVs available for release, mislocalization of endophilin-A, and altered presynaptic localization of clathrin light chain. Furthermore, reduction of endophilin-A affected expression levels of active zone proteins. Collectively, these findings redefine the role of CAST/ELKS beyond active zone structural organization, demonstrating that their interaction with endophilin-A contributes to maintaining the SV pool required for sustained synaptic transmission at the presynaptic active zone.

## Teaser

Mori et al. demonstrate that the presynaptic active zone proteins CAST and ELKS directly interact with endophilin-A to maintain the functional synaptic vesicle pool. This study redefines their roles beyond structural organization, highlighting how they coordinate synaptic vesicle release processes necessary for efficient neurotransmission.

## Introduction

The active zone, a dense molecular structure located at presynaptic terminals, plays a pivotal role in regulating key neuronal processes such as calcium influx, synaptic vesicle (SV) tethering and fusion, and maintenance of the readily releasable pool (RRP) of SVs. SVs play a central role in synaptic transmission through the storage and release of various neurotransmitters. Local recycling at the nerve terminal enables the refilling of SVs with neurotransmitters to maintain neurotransmission. The active zone is organized by the active zone scaffolding proteins, including Munc13, Rim1, CAST/ELKS, RimBP, Bassoon/Piccolo, and Liprin. These proteins interact to coordinate the release machinery and ensure efficient synaptic transmission (Südhof, 2012). For example, Munc13 regulates SV priming and syntaxin-1 localization (Sakamoto et al., 2018), while Rim1 and RimBP primarily control the localization and function of calcium channels (Kaeser et al., 2011). Among these components, CAST and ELKS play a role as scaffolding proteins, linking key active zone components such as Bassoon/Piccolo, Rim, and Munc13 (Ohtsuka et al., 2002; Takao-Rikitsu et al., 2004).

SVs at the presynaptic terminal show varying responses to stimuli. To account for these differences, a model has been proposed that categorizes SVs into distinct pools based on their stimulus responsiveness (Rizzoli and Betz, 2005; Alabi and Tsien, 2012). Broadly, SVs are divided into two main pools: the total recycling pool (TRP), which actively recycles during stimulation, and the resting pool (RP), which remains less responsive. Within the TRP, a smaller subset known as the RRP responds almost instantaneously to stimuli. For example, in hippocampal neurons, the RRP constitutes ∼5% of the total SV pool, while the TRP comprises 40–50%, and the RP accounts for 50–60% (Rizzoli and Betz, 2005; Alabi and Tsien, 2012). Although the RRP is typically thought to consist of SVs directly in contact with the active zone, some evidence suggests that SVs not in direct contact may also contribute to release, a hypothesis that remains a subject of ongoing debate (Kaeser and Regehr, 2017). The ratio of TRP to RP can vary depending on the neuronal state. For instance, inhibition of CDK5 kinase has been shown to increase the proportion of the TRP (Kim and Ryan, 2010), highlighting the dynamic regulation of SV pool distribution in response to cellular conditions.

Efficient neurotransmission depends on the coordinated regulation of exocytosis and endocytosis to ensure the availability of SVs for continuous recycling. At the presynaptic terminal, exocytosis is triggered by calcium influx following action potential stimulation, leading to the release of neurotransmitters into the synaptic cleft. To sustain neurotransmission, SV membranes and proteins, such as synaptophysin and VAMP-2/synaptobrevin, are retrieved near the active zone (within 50–300 nm) through a process called endocytosis (Hua et al., 2011; Watanabe et al., 2018). Current models of SV membrane retrieval involve either clathrin-mediated endocytosis or clathrin-independent endocytosis (Kononenko and Haucke, 2015). Proteins critical for SV recycling include clathrin and core clathrin coat components such as AP-2 (Kononenko et al., 2014), as well as BAR domain–containing proteins, like endophilin-A, which can sense bilayer curvature (Bai et al., 2010; Milosevic et al., 2011). Additionally, proteins involved in phosphoinositide metabolism, such as synaptojanin-1, and membrane fission proteins, such as dynamin, play essential roles in the endocytic process (Saheki and De Camilli, 2012). After endocytosis, SVs are re-acidified, and neurotransmitters (e.g., glutamate and GABA) are reloaded to restore functional SVs. Exocytosis and endocytosis are tightly coupled processes, referred to as exo- and endocytosis coupling, which is essential for maintaining SV recycling and ensuring adequate pool size (Haucke et al., 2011; Maritzen and Haucke, 2018). However, the precise mechanisms underlying pool size regulation remain unclear despite decades of research.

To ensure SV retrieval occurs rapidly and in synchronization with SV release, exo- and endocytosis coupling likely involves a series of feedback mechanisms. The roles of certain proteins in this process are well established (e.g., SNARE proteins in exocytosis and clathrin and dynamin in endocytosis), yet the signaling pathways or proteins that link exocytosis to endocytosis are not fully elucidated. Recent work has shown that endophilin-A plays an important role in maintaining the replacement SV pool, in addition to its established function in endocytosis (Ogunmowo et al., 2025). These findings suggest that endophilin-A also contributes to release-related processes beyond its well-known role in endocytosis. Therefore, in this study, we sought to define the role of CAST/ELKS in maintaining the size of the TRP of SVs and regulating SV dynamics. We demonstrated that CAST/ELKS interact with endophilin-A and that disruption of this interaction led to endophilin-A mislocalization, defective SV release, and altered presynaptic distribution of clathrin light chain (CLC). Additionally, an endophilin-A mutant with dynamin dose dependently reduced the binding affinity to CAST, impaired SV release, and destabilized active zone proteins, including CAST and Rim1. These findings revealed that the interaction of CAST/ELKS plays a critical role in the endophilin-A–dependent maintenance of SV pool size. Better understanding of the molecular mechanisms of maintaining SV pool size not only deepens our understanding of sustained neurotransmission but may lead to therapeutic targets for neurodegenerative and psychiatric conditions.

## Results

### CAST/ELKS regulate neurotransmitter release

**Figure 1.**
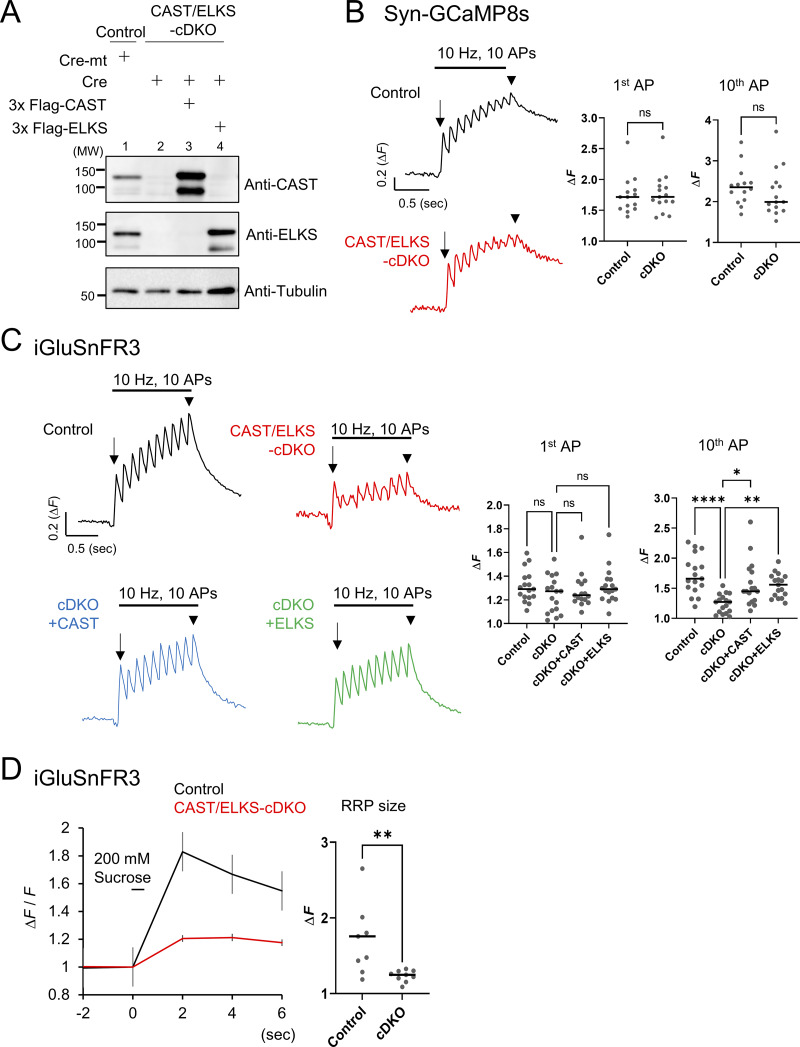
**Measurement of calcium concentration at presynaptic terminal and glutamate release in CAST/ELKS-cDKO hippocampal neurons. (A)** CAST/ELKS protein expression level in mouse hippocampal neurons from CAST-flox/ELKS-flox mouse. CAST, ELKS, and tubulin were detected by the western blot analysis in control (Cre-mutant), CAST/ELKS-cDKO (Cre), CAST/ELKS-cDKO with CAST (Cre with the short CaMKII promoter, Ca0.3-derived CAST), and CAST/ELKS-cDKO with ELKS (Cre with Ca0.3-derived ELKS). **(B)** Calcium concentration at presynaptic terminal in CAST/ELKS cDKO neurons. Activity-dependent Syn-GCaMP8s (synaptophysin-GCaMP8s) fluorescence response in control (black) or CAST/ELKS-cDKO (red) mouse hippocampal cultured neurons. The left traces show the average fluorescence of individual Syn-GCaMP8s upon a 10 Hz, 10 AP stimulation. Results are mean ± SEM. *n* = 15 images, each. The points for the 1st AP and 10th AP are indicated by arrows and arrowheads, respectively. The right dot plots show the 1st AP (right) and the 10th AP data (left). P values were obtained by nonparametric Mann–Whitney test. **(C)** Released glutamate in CAST/ELKS-cDKO neurons. Activity-dependent iGluSnFR3 fluorescence response in control (black), CAST/ELKS-cDKO (red), CAST/ELKS-cDKO with CAST (blue), or CAST/ELKS-cDKO with ELKS (green) in hippocampal neurons. The left traces show average fluorescence of individual iGluSnFR3 upon the 10th AP stimulation (10 Hz). Results are mean ± SEM. *n* = 15 images, each. The points for the 1st AP and 10th AP are indicated by arrows and arrowheads, respectively. The right dot plots show results from the 1st AP (right) and 10th AP (left). P values were obtained by comparison with cDKO neurons (one-way ANOVA with post hoc Bonferroni’s multiple comparison test, *P < 0.05, **P < 0.01, ****P < 0.0001). **(D)** RRP size in CAST/ELKS cDKO neurons. The sucrose solution mediated iGluSnFR3 fluorescence response in control (black), CAST/ELKS-cDKO (red) in the hippocampal neurons. The left trace shows average fluorescence of individual iGluSnFR3 upon 250 mM sucrose solution stimulation. Results are mean ± SEM. *n* = 10 images, each. The right dot plot shows the increase in fluorescence of iGluSnFR3 in response to 250 mM sucrose solution for each image. P values were obtained by nonparametric Mann–Whitney test, **P < 0.01. Source data are available for this figure: SourceData F1.

To investigate the role of CAST and ELKS in calcium dynamics, glutamate release, and SV recycling, we generated CAST/ELKS-conditional double knockout (cDKO) hippocampal neurons. This was achieved by transducing adeno-associated virus (AAV) expressing Cre recombinase under the CAG promoter into hippocampal neurons from *CAST*-flox/*ELKS*-flox mice (Hagiwara et al., 2018). Immunoblotting confirmed that CAST and ELKS protein levels were undetectable in cDKO neurons (Fig. 1 A). First, we examined presynaptic calcium concentrations using Syn-GCaMP8s, a calcium sensor fused with the cytoplasmic region of synaptophysin and jGCaMP8s (Dreosti et al., 2009; Brockmann et al., 2020; Zhang et al., 2023). Syn-GCaMP8s fluorescence showed no significant difference between control neurons and CAST/ELKS-cDKO neurons, regardless of stimulation with the first (1st AP) or tenth action potential (10th AP) (Fig. 1 B). These findings suggest that CAST and ELKS do not significantly affect the presynaptic calcium concentration in hippocampal neurons.

Next, we measured glutamate release using iGluSnFR3, a genetically encoded glutamate sensor (Aggarwal et al., 2023). The fluorescence response to the 1st AP was numerically (but not significantly) reduced in CAST/ELKS-cDKO neurons, compared with control neurons (Fig. 1 C), indicating minimal involvement of CAST and ELKS in the initial SV fusion process. In contrast, the fluorescence response after the 10th AP was significantly reduced in CAST/ELKS-cDKO neurons, compared with control neurons, while exogenous expression of CAST or ELKS rescued this reduction in CAST/ELKS-cDKO neurons (Fig. 1 C). This result suggests that CAST and ELKS play a role in sustaining neurotransmitter release during prolonged stimulation in hippocampal excitatory neurons, likely reflecting a reduction in RRP size (Held et al., 2016; Wang et al., 2016). To examine this, we determined the RRP size by applying a sucrose solution and measuring iGluSnFR3 fluorescence in excitatory neurons (Fig. 1 D). This confirmed a reduction in RRP size in CAST/ELKS-cDKO neurons. Together, these findings support a role for CAST and ELKS in both sustaining neurotransmitter release and maintaining RRP size.

### CAST/ELKS maintain SV properties

**Figure 2.**
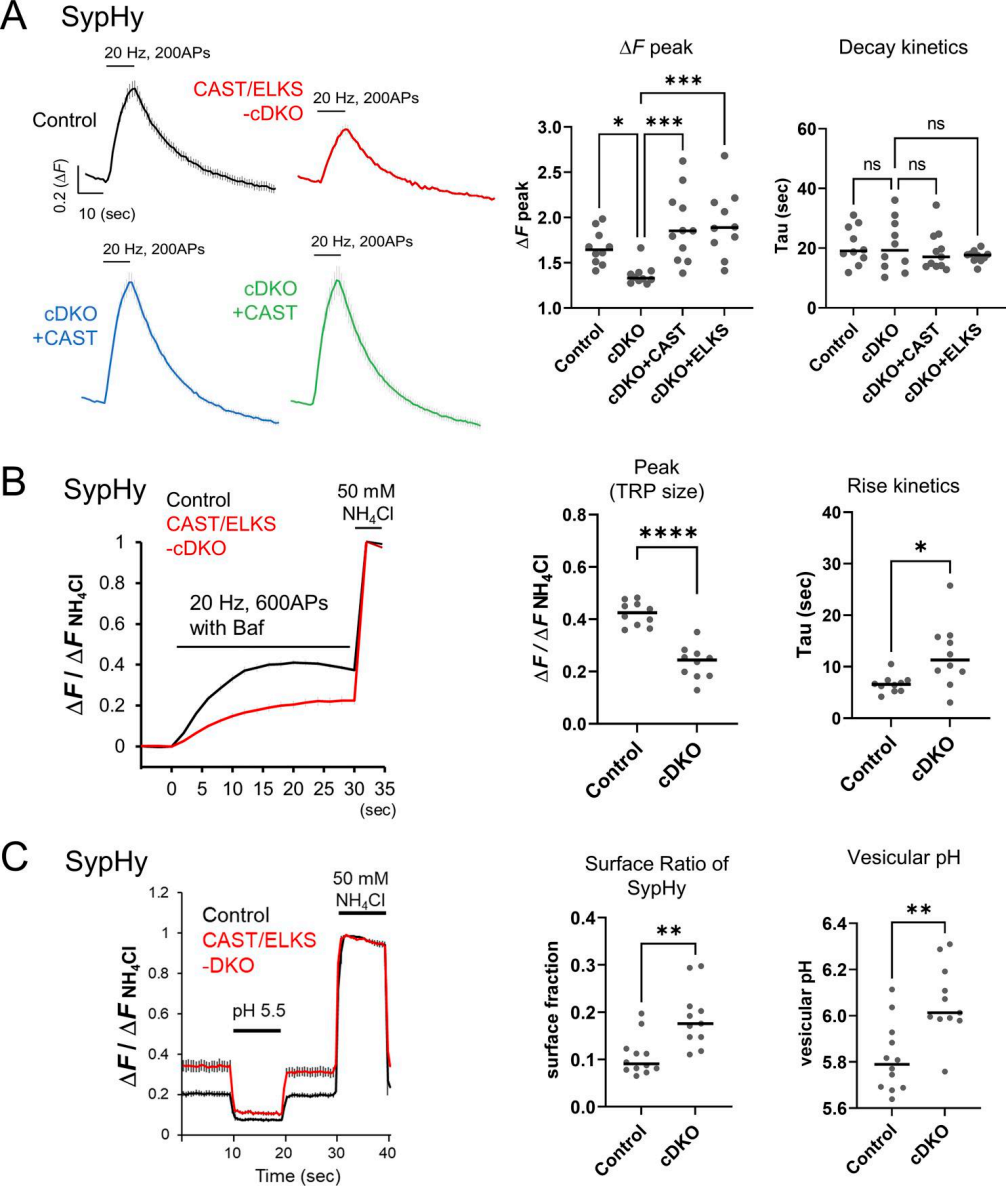
**Measurement of the SV recycling process in CAST/ELKS-cDKO hippocampal neurons. (A)** SV release and endocytosis kinetics in CAST/ELKS cDKO neurons. Activity-dependent SypHy fluorescence response in control (black), CAST/ELKS-cDKO (red), CAST/ELKS-cDKO with CAST (blue), or CAST/ELKS-cDKO with ELKS (green) in mouse hippocampal cultured neurons. Left traces show average fluorescence of individual SypHy upon 20 Hz stimulation with 200 APs. Results are mean ± SEM. *n* = 10 images, each. Right dot plots show the peak of 200 APs (left) and the decay constant (tau) after stimulation (right). P values were obtained by comparison with cDKO neurons (one-way ANOVA with post hoc Bonferroni’s multiple comparison test, *P < 0.05, ***P < 0.001). **(B)** TRP size and SV replenishment kinetics in CAST/ELKS-cDKO neurons. Left traces show the TRP size in control (black) or CAST/ELKS-cDKO (red) neurons. Results are mean ± SEM. *n* = 15 images, each. Right dot plots show the peak of 600 APs (left) and the rise constant (tau) during stimulation (right). P values were obtained by nonparametric Mann–Whitney test, *P < 0.05, ****P < 0.0001. **(C)** Surface ratio of SypHy and vesicular pH of SypHy-bearing vesicles in the CAST/ELKS cDKO neurons. Left traces show the average for estimating the surface fraction of SypHy and vesicular pH in control (black) or CAST/ELKS-cDKO (red) neurons. Results are mean ± SEM. *n* = 10 images, each. Right dot plots show quantification of the surface fraction ratio of SypHy (right) and vesicular pHs (left). P values were obtained by nonparametric Mann–Whitney test, **P < 0.01.

**Figure S1.**
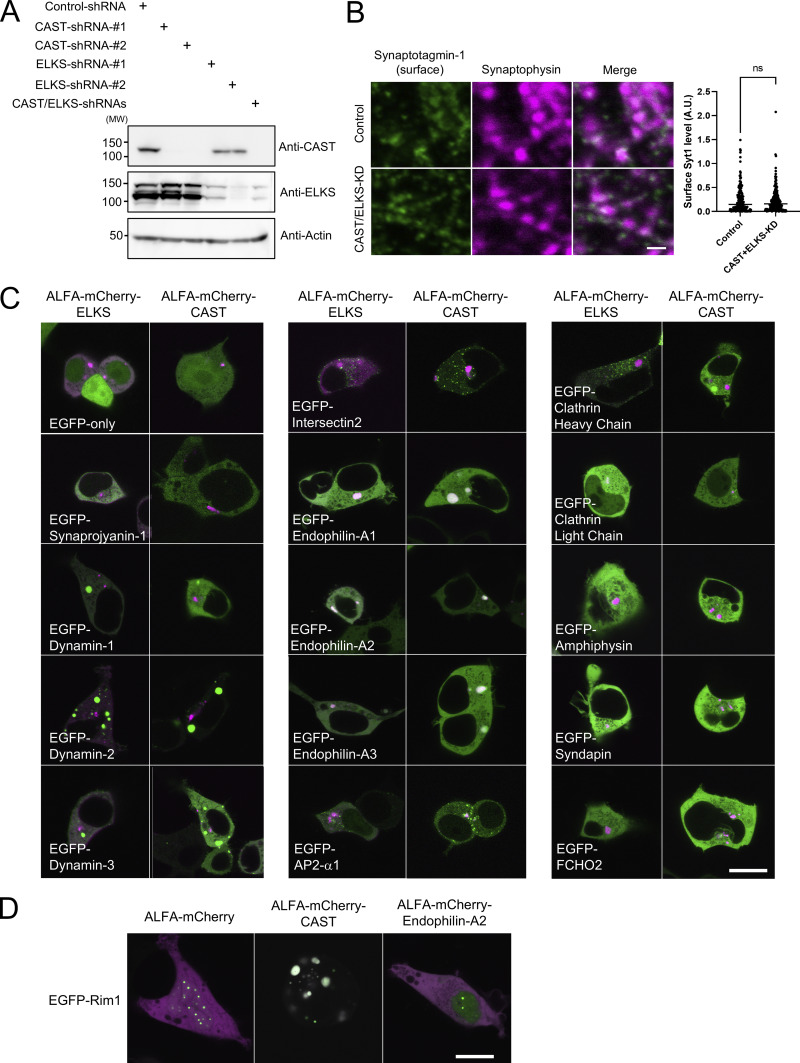
**Surface level of endogenous SV protein in CAST/ELKS-KD neurons and a screening analysis of co-condensation between CAST/ELKS and endocytosis-related proteins in the HEK cells. (A)** CAST, ELKS, and tubulin were detected by western blot analysis in rat hippocampal neurons expressing control-shRNA, CAST-shRNA-#1, CAST-shRNA-#2, ELKS-shRNA-#1, ELKS-shRNA-#2, or CAST/ELKS-shRNAs. CAST/ELKS double-knockdown (CAST/ELKS-KD) neurons were generated by co-expression of CAST-shRNA-#1, CAST-shRNA-#2, ELKS-shRNA-#1, and ELKS-shRNA-#2. **(B)** Surface levels of endogenous synaptotagmin-1 in CAST/ELKS-KD neurons. The left panels show surface-exposed synaptotagmin-1 (green) and total synaptophysin (magenta) in control neurons (upper panels) and CAST/ELKS-KD neurons (lower panels). Scale bar, 2 μm. The dot plots on the right show quantification of the surface synaptotagmin-1 fluorescence intensity (control and CAST/ELKS-KD; *n* = 225 puncta from 12 images). P values were calculated using the nonparametric Mann–Whitney test. **(C)** Screening proteins that of co-accumulate with ALFA-mCherry-CAST or ALFA-mCherry-ELKS (magenta), and EGFP-tagged endocytosis-related proteins including synaptojanin-1, dynamin-1, dynamin-2, dynamin-3, intersectin2, endophilin-A1, endophilin-A2, endophilin-A3, AP2-α, CHC, CLC, amphiphysin, syndapin, and FCHO2 (green). Scale bar: 10 μm. **(D)** EGFP-RIM1 result in droplet-like condensate with ALFA-mCherry-CAST (middle panel), but not condensate with ALFA-mCherry-endophilin-A2. Scale bar: 10 μm. Source data are available for this figure: SourceData FS1.

To investigate the SV recycling process in CAST/ELKS-cDKO neurons, we used SypHy, a pH-sensitive fluorescent protein fused to the intraluminal region of synaptophysin (Granseth et al., 2006), which measures the endocytic behavior of SVs. Compared with control neurons, we observed a ∼50% reduction in SypHy fluorescence increase following 200 repetitive stimulations in CAST/ELKS-cDKO neurons, although the decay kinetics after stimulation remained unchanged (Fig. 2 A). This reduction in the fluorescence increase was rescued by expression of either CAST or ELKS (Fig. 2 A). This reduction in fluorescence increase could result from either: (1) a reduction in TRP size or (2) a slower SV replenishment rate. To distinguish these possibilities, we performed 600 prolonged stimulations in the presence of the proton pump inhibitor, bafilomycin. This blocked re-acidification and enabled the estimation of both TRP size and replenishment rate (Mori et al., 2021). We found that TRP size was reduced by ∼50% in CAST/ELKS-cDKO neurons, along with a significant decrease in the replenishment rate. However, the reduction in TRP size was more pronounced (Fig. 2 B). These findings indicate that CAST and ELKS are crucial for maintaining both TRP size and the replenishment rate. Additionally, we analyzed the surface fraction of SypHy under basal conditions and the intraluminal pH of SypHy-containing vesicles using acid quenching and ammonium chloride solution (Mori et al., 2021). CAST/ELKS-cDKO neurons showed increased surface SypHy levels and a higher intraluminal pH compared with control neurons (Fig. 2 C). Based on SypHy measurements, overexpression increased signaling at the cell surface; however, we did not observe a corresponding accumulation of endogenous SV proteins, such as synaptotagmin-1, on the plasma membrane (Fig. S1, A and B). These results suggest that CAST/ELKS does not play a major role in basal endocytosis but instead influences the intravesicular pH.

### CAST/ELKS interact with endophilin-A

**Figure 3.**
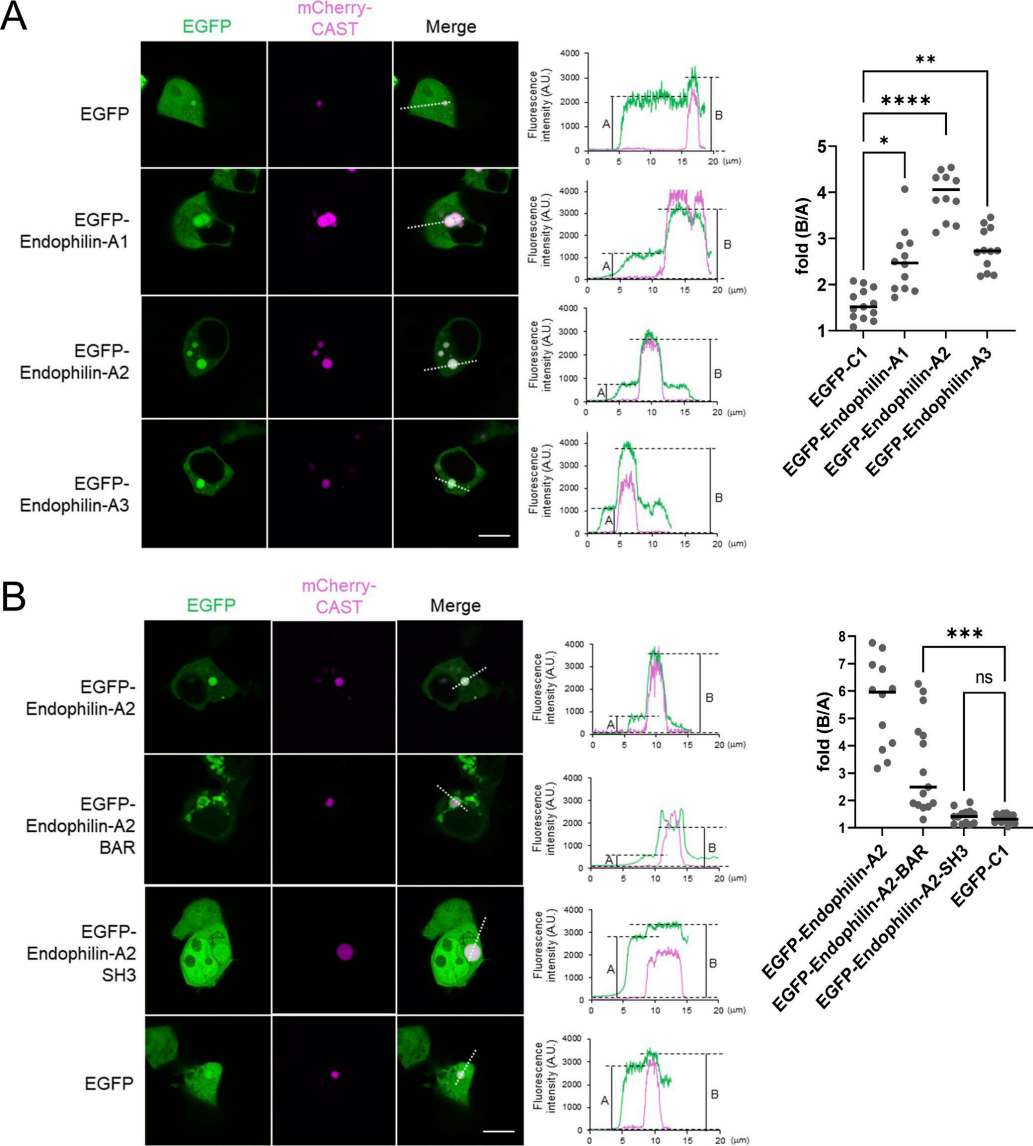
**Endophilin-A co-condensates with CAST/ELKS in HEK293T cells. (A and B)** EGFP-endophilin-A1, -A2, or -A3 expressed with mCherry-CAST resulted in co-condensates in the HEK cells. EGFP alone with mCherry-CAST (upper images) did not result in condensate structures. The dotted line indicates a line scan of each fluorescence protein. Right dot plots show the ratio of average fluorescence intensity at the condensate (B) to cytosolic region (A) Scale bar: 10 μm. *n* = 10 cells, each. P values were obtained by comparison with EGFP-C1–expressing cells (one-way ANOVA with post hoc Bonferroni’s multiple comparison test, *P < 0.05, **P < 0.01, ****P < 0.0001). **(B)** EGFP–endophilin-A2 and EGFP–endophilin-A2-BAR expressed with mCherry-CAST resulted in condensates. EGFP–endophilin-A2-SH3 or EGFP alone expressed with mCherry-CAST did not result in condensates. The dotted line indicates a line scan of each fluorescence protein. Right dot plots show the ratio of average fluorescence intensity at the condensate (B) to cytosolic region (A). Scale bar: 10 μm. *n* = 10 cells, each. P values were obtained by comparison with EGFP–endophilin-A2–expressing cells (one-way ANOVA with post hoc Bonferroni’s multiple comparison test, ***P < 0.001).

To investigate the involvement of CAST/ELKS in SV recycling processes, including the maintenance of the SV pool size, we hypothesized that CAST/ELKS are involved in the process of endocytosis itself or the process of SV regeneration that occurs after endocytosis. To test this, we comprehensively screened endocytosis-related proteins that interact with CAST and ELKS. Previous studies have shown that CAST and ELKS form co-condensates with their interacting proteins, such as Rim1, when co-expressed in the HEK cells (Ohtsuka et al., 2002). We extended this work by co-expressing CAST or ELKS with a panel of endocytosis-related proteins in the HEK cells, including synaptojanin-1, dynamin isoforms, intersectin-2, endophilin-A (A1, A2, and A3), AP2-α, clathrin heavy chain (CHC), CLC, amphiphysin, syndecan, and FCHO2. Among these, only EGFP–endophilin-A1, -A2, and -A3 formed co-condensates with ALFA-mCherry-CAST and ALFA-mCherry-ELKS (Fig. S1 C), with the highest interaction efficiency observed for EGFP–endophilin-A2 (Fig. 3 A). This is consistent with a previous study identifying endophilin-A1 and endophilin-A2 as neighboring proteins of CAST and Rim1 in the mouse midbrain (Kershberg et al., 2022), although Rim1 did not form co-condensates with endophilin-A in the HEK cells (Fig. S1 D), suggesting a unique interaction between CAST/ELKS and endophilin-A. Additionally, given that endophilin-A has previously been reported to be required for the maintenance of SV pools (Ogunmowo et al., 2025), it represents a reasonable candidate for mediating the CAST/ELKS-dependent mechanism that maintains vesicle pool size.

**Figure S2.**
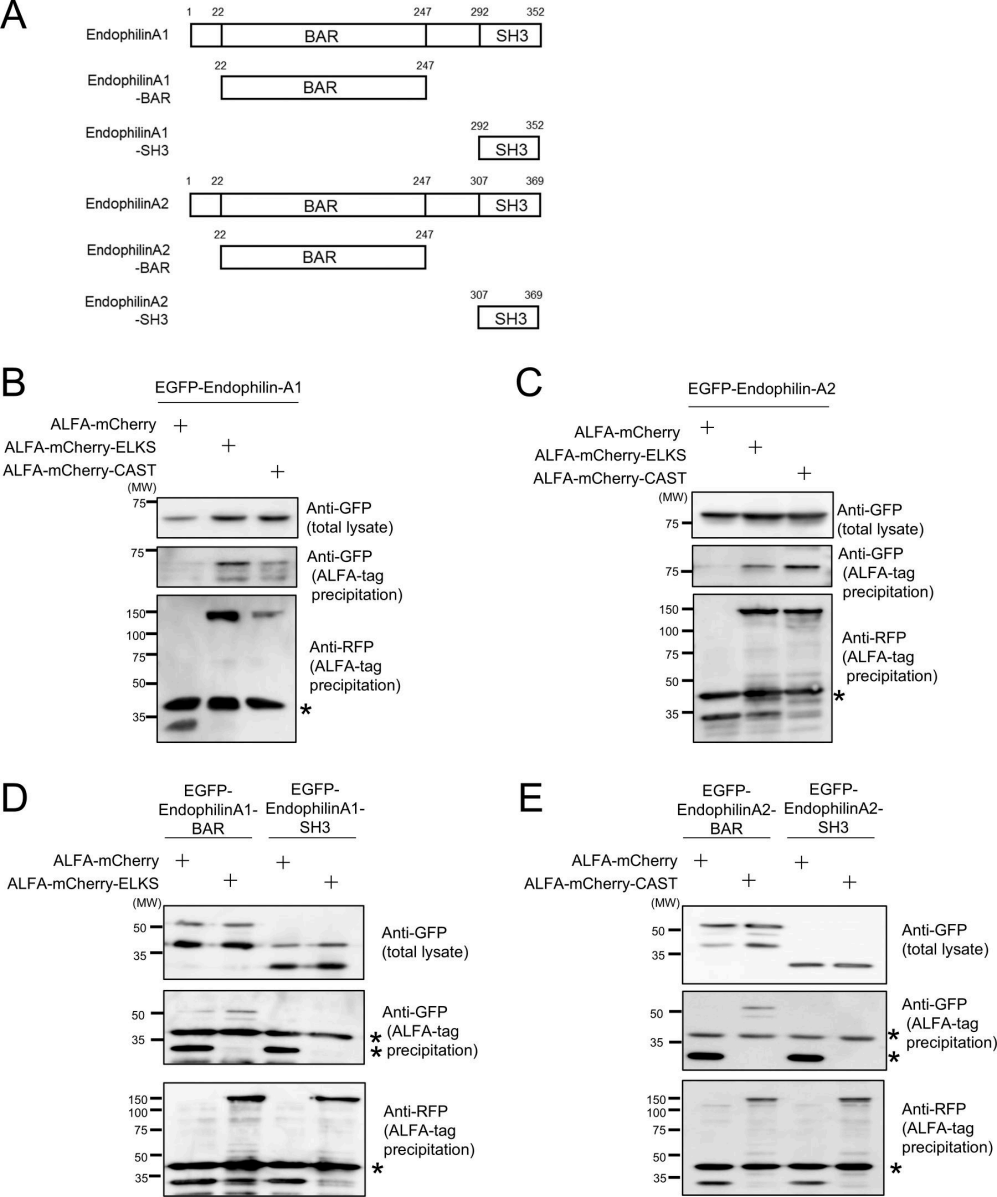
**Physiological interaction of CAST/ELKS and endophilin-A in the HEK cells. (A)** Cartoon image of the deletion constructs of endophilin-A1 and endophilin-A2. **(B and C)** Binding of CAST or ELKS to endophilin-A1 (A), or endophilin-A2 (B). HEK cells expressing EGFP-endophilin-A1 were precipitated with ALFA-mCherry, ALFA-mCherry-ELKS, or ALFA-mCherry-CAST with recombinant ALFA nanobody-GST protein-binding GSH-Sepharose. Precipitated EGFP–endophilin-A1 or EGFP–endophilin-A2 was detected by western blot analysis. **(D and E)** BAR domain of endophilin-A1– (D) or endophilin-A2– (E) mediated binding of CAST. HEK cells expressing EGFP–endophilin-A1-BAR or EGFP–endophilin-A1-SH3, or EGFP–endophilin-A2-BAR or EGFP–endophilin-A2-SH3 was precipitated with ALFA-mCherry, ALFA-mCherry-ELKS, or ALFA-mCherry-CAST with recombinant ALFA nanobody-GST protein-binding GSH-Sepharose. Precipitated EGF–endophilin-A1 or EGFP–endophilin-A2 were detected by western blot analysis. Source data are available for this figure: SourceData FS2.

To confirm this interaction, we performed immunoprecipitation assays using the HEK cells, which showed that both ALFA-mCherry-CAST and ALFA-mCherry-ELKS coprecipitated with EGFP–endophilin-A1 (Fig. S2, A and B) and EGFP–endophilin-A2 (Fig. S2, A and C). As the results showed that CAST and ELKS bind similarly to endophilin-A, we performed the rest of these experiments using CAST as a representative. We next sought to determine the specific region of endophilin-A responsible for the interaction with CAST/ELKS. Endophilin-A contains two key domains: a BAR domain that recognizes curved lipid bilayers (Farsad et al., 2001) and an SH3 domain that binds endocytosis-related proteins such as dynamin-1 (Ringstad et al., 2001). Using deletion mutants, we found that although full-length endophilin-A2 formed co-condensates with ALFA-mCherry-CAST, the SH3 domain alone did not (Fig. 3 B). In contrast, the BAR domain alone still formed co-condensates, albeit with lower efficiency compared with the full-length protein, indicating that the BAR domain is crucial for the interaction. Immunoprecipitation further confirmed these findings, showing that the BAR domain but not the SH3 domain, coprecipitated with CAST and ELKS (Fig. S2, A, D, and E).

**Figure 4.**
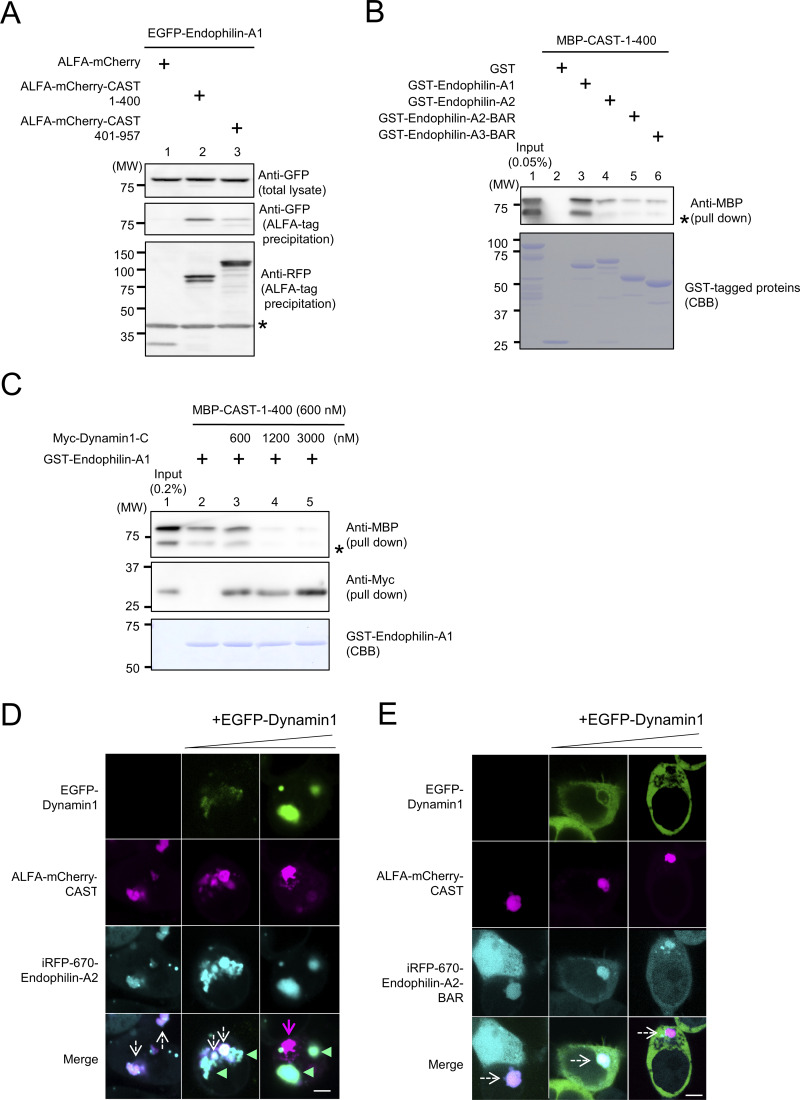
**Dynamin-mediated inhibition of CAST and endophilin-A binding. (A)** N-terminal region of CAST binds to endophilin-A1. HEK293T cells expressing EGFP–endophilin-A1 were precipitated with ALFA-mCherry, ALFA-mCherry-CAST-1-400, or ALFA-mCherry-CAST-401-957 with recombinant ALFA nanobody-GST protein-binding GSH-Sepharose. Precipitated EGFP–endophilin-A1 was detected by western blot analysis. **(B)** Direct binding of CAST to endophilin-A family proteins. Recombinant protein of MBP-CAST-1-400 was precipitated with GST-endophilin-A1-full, GST-endophilin-A2-full, GST-endophilin-A2-BAR, or GST-endophilin-A3-BAR. MBP-CAST-1-400 was detected by western blot analysis and GST-endophilin-A proteins were detected by Coomassie brilliant blue (CBB) staining. **(C)** Dynamin-mediated inhibition of CAST and endophilin-A binding. MBP-CAST-1-400 and different amounts of Myc-dynamin-1-C (residues 657–867) were precipitated with GST-endophilin-A1-full. MBP-CAST-1-400 and Myc-dynamin-1-C were detected by western blot analysis and GST-endophilin-A1-full was detected by CBB staining. **(D)** HEK293T cells were transfected with EGFP-dynamin-1 (top panels; left panels; none of DNA; middle panels 0.01 μg of DNA; right panels 0.1 μg of DNA), ALFA-mCherry-CAST (second panels from the top), and iRFP670–endophilin-A2-expressing plasmid DNA (third panels from the top). Merged images show bottom panels, and white dotted arrow indicates co-condensation of ALFA-mCherry-CAST and iRFP670-endophilin-A2; green arrowhead indicates co-condensation of EGFP–dynamin-1 and iRFP670–endophilin-A2, and magenta arrow indicates condensation of ALFA-mCherry-CAST. Scale bar: 10 μm. **(E)** HEK293T cells were transfected with EGFP–dynamin-1 (top panels; left panels; none of DNA; middle panels 0.01 μg of DNA; right panels 0.1 μg of DNA), ALFA-mCherry-CAST (second panels from the top), and iRFP670–endophilin-A2 BAR domain–expressing plasmid DNA (third panels from the top). Merged images show bottom panels, and white dotted arrow indicates co-condensation of ALFA-mCherry-CAST, and iRFP670–endophilin-A2 BAR domain. Scale bar: 10 μm. Source data are available for this figure: SourceData F4.

Next, we examined the interaction between recombinant CAST (N-terminal region) (Kiyonaka et al., 2012) and endophilin-A. In the HEK cells, the N-terminal region of CAST (residues 1–400) showed stronger binding to endophilin-A compared with the C-terminal region (residues 401–957) (Fig. 4 A). Recombinant CAST1-400 also bound to full-length endophilin-A1, -A2, and the BAR domains of endophilin-A2 and -A3 (Fig. 4 B), confirming the specific interaction of CAST with the BAR domain of endophilin-A family proteins. Because endophilin-A binds dynamin-1 via its SH3 domain (Ringstad et al., 2001), we examined whether CAST/ELKS, endophilin-A, and dynamin-1 form a ternary complex or whether dynamin-1 is not involved in the CAST/ELKS–endophilin-A complex. Binding assays with recombinant proteins showed that increasing concentrations of the C-terminal region of dynamin-1 (residues 657–868) reduced the amount of CAST bound to endophilin-A, while dynamin-1 binding to endophilin-A increased (Fig. 4 C). This finding suggests that dynamin-1 acts specifically on the CAST–endophilin-A complex, removing CAST and thereby enabling dynamin-1 to bind to endophilin-A. Supporting this, co-expression of endophilin-A2, dynamin-1, and CAST in the HEK cells showed that dynamin-1 formed co-condensates with endophilin-A2, while CAST did not (Fig. 4 D). However, when only the BAR domain of endophilin-A2 was expressed, CAST formed co-condensates with endophilin-A2-BAR, whereas dynamin-1 did not (Fig. 4 E). This indicates that CAST binds to the BAR domain of endophilin-A, while dynamin-1 binds to the SH3 domain. Thus, binding of dynamin-1 and CAST to endophilin-A appears to be domain-specific and mutually exclusive.

### CAST and endophilin-A form liquid–liquid phase separation like-condensates in HEK293T cells

**Figure 5.**
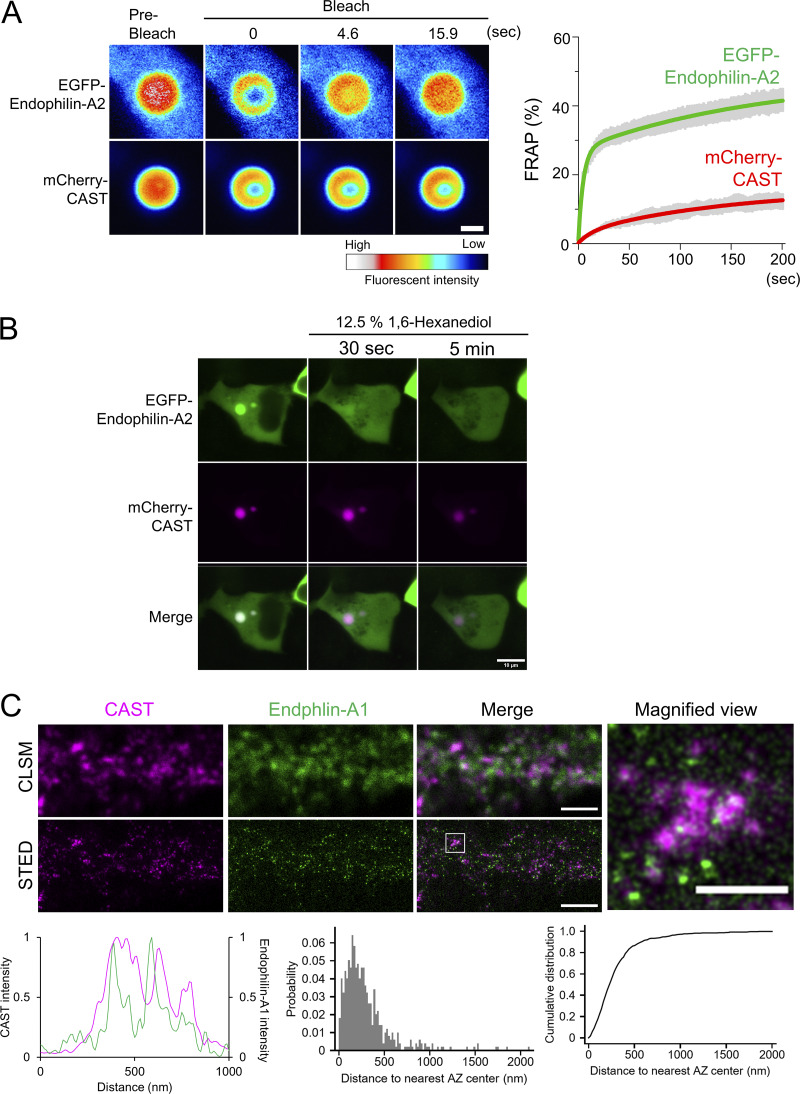
**CAST–endophilin condensates exhibit liquid-like behavior and localize near active zones. (A)** FRAP analysis of liquid condensates in HEK293T cells co-transfected with mCherry-CAST and EGFP–endophilin-A2. The right panels show representative images before and after photobleaching. Scale bar, 2 μm. The left graph shows fluorescence recovery curves for mCherry-CAST (*n* = 28, red line) and EGFP–endophilin-A2 (*n* = 28, green line). Error bars represent the SEM. **(B and C)** Effect of 1,6-hexanediol treatment on CAST–endophilin-A assemblies. HEK293T cells co-transfected with mCherry-CAST and EGFP–endophilin-A2 were treated with 12.5 % 1,6-hexanediol at the indicated time points. Scale bar; 10 μm (C) Immunofluorescence images of CAST (magenta) and endophilin-A1 (green) in cultured rat hippocampal neurons. The upper left panels show confocal laser scanning microscopy (CLSM) images and corresponding STED images. The upper right panel presents an enlarged view of the region indicated by a white box in the STED image. Arrows indicate sites where CAST and endophilin-A1 co-localize. Scale bars: 2 µm for the original images and 500 nm for the enlarged image. The bottom left panel shows fluorescence intensity profiles from the enlarged STED images. The bottom middle and right panels show a histogram and cumulative distribution, respectively, of the distances from individual endophilin-A1 puncta to the nearest active zone (AZ) center (*n* = 497 puncta from three independent cultures).

Endophilin-A has been reported to transiently interact with endocytosis-related and presynaptic proteins through liquid–liquid phase separation (LLPS) (Mondal et al., 2022; Yoshida et al., 2023). Moreover, heterologous expression in HEK293T cells has been used to characterize phase separation behavior of synaptic proteins and to dissect the molecular mechanisms underlying condensate formation (Hoffmann et al., 2023; Ogunmowo et al., 2025). Based on this observation, we investigated whether the CAST–endophilin-A condensates observed in the HEK cells represent LLPS. First, we examined the dynamics of CAST and endophilin-A using FRAP analysis. Both CAST and endophilin-A2 showed clear FRAP, indicating dynamic molecular exchange within the condensates (Fig. 5 A). These results indicate that the condensates formed by CAST and endophilin-A exhibit LLPS-like properties. The fluorescence recovery time of endophilin-A2 was shorter than that for CAST, suggesting that endophilin-A2 exchanges more rapidly within the condensates than CAST. Furthermore, when 1,6-hexanediol, an inhibitor of LLPS formation, was added, endophilin-A2 separated from the CAST–endophilin-A co-condensates (Fig. 5 B). Together, these results support a phase-separation-based mechanism underlying the formation of CAST–endophilin-A condensates in the HEK cells.

### Localization of CAST and endophilin-A at the presynaptic terminal

In previous reports, using stimulated emission depletion (STED) microscopy, a subset of endophilin-A1 puncta localizes between synapsin1-positive vesicle clusters and the active zone protein Bassoon, suggesting that endophilin-A1 functions within or near the active zone (Ogunmowo et al., 2025). To further define its spatial relationship with active zone components, we examined the proximity of CAST to endophilin-A1. We performed STED microscopy to resolve the nanoscale localization of CAST and endophilin-A1. STED imaging and subsequent quantification of the distances from endophilin-A1 puncta to the midline of CAST signals, using histogram and cumulative frequency analysis, indicated that endophilin-A1 puncta are positioned within or adjacent to CAST signals (Fig. 5 C). These results demonstrate partial nanoscale colocalization of endophilin-A1 with CAST and support a spatial association between these proteins at presynaptic sites.

### CAST/ELKS–endophilin-A and SV release

**Figure 6.**
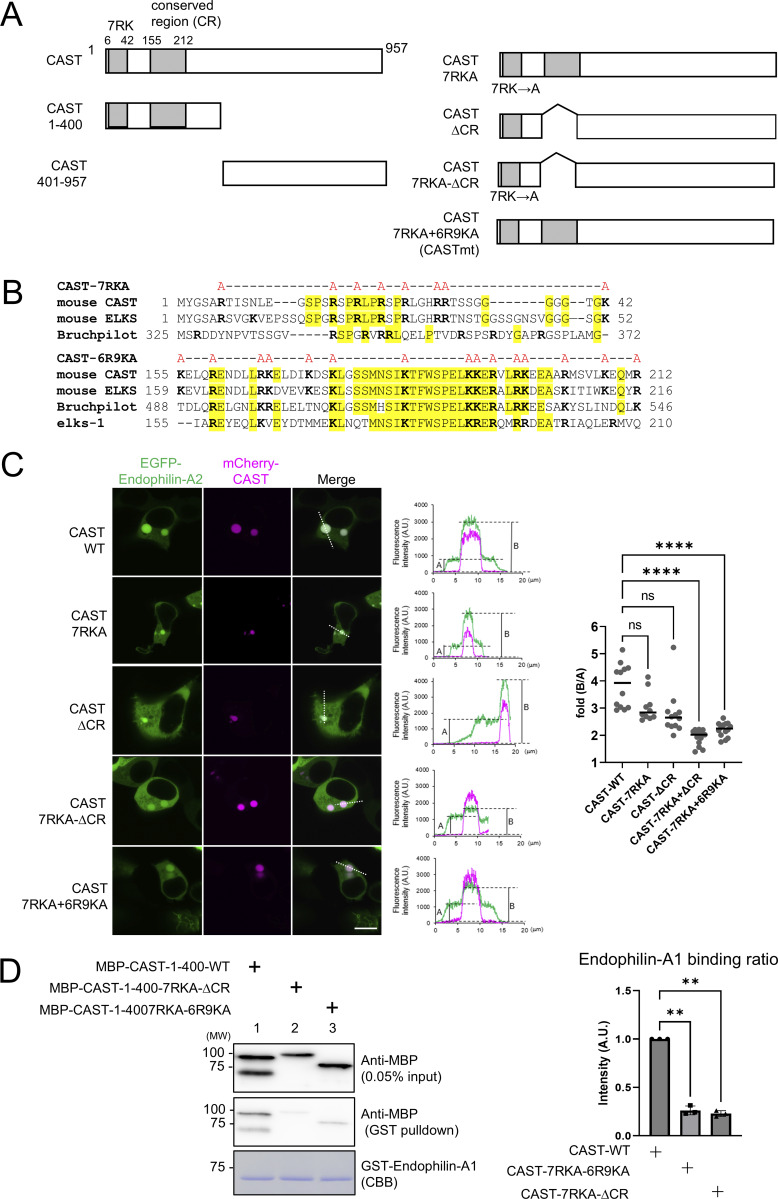
**Determining the amino acid region of CAST that binds to endophilin-A. (A)** Cartoon image of the deletion and mutation constructs of CAST. **(B)** The regions of the amino acids sequence that have been evolutionarily conserved in CAST. The amino acids 6–42 of mouse CAST shown in the upper panel are partially homologous to Bruchpilot, the ortholog of *Drosophila*. The 155–212 amino acids of mouse CAST shown in the panel below are partially homologous not only to Bruchpilot but also to elks-1, a homologous molecule in *C. elegans*. **(C)** EGFP–endophilin-A2 expressed with mCherry-CAST, CAST-7RKA, or CAST-ΔCR results in condensates, which are rarely observed with CAST-7RKA+ΔCR and CAST-7RKA+6R9KA. The dotted line indicates a line scan of each fluorescence protein. Right dot plots show the ratio of average fluorescence intensity at the condensate (B) to cytosolic region (A). Scale bar: 10 μm. *n* = 10 cells, each. P values were obtained by comparison with CAST-WT (one-way ANOVA with post hoc Bonferroni’s multiple comparison test, ****P < 0.0001). **(D)** Decrease the affinity of mutant CAST for endophilin-A-1. MBP-CAST-1-400, MBP-CAST-1-400-7RK+6R9KA, or MBP-CAST-1-400-7RK+ΔCR were precipitated with GST–endophilin-A1-full. MBP-CAST-1-400 was detected by the western blot analysis and GST–endophilin-A1-full was detected by the CBB staining. Right bar graph shows quantification of the left bands (*n* = 3 independently prepared samples). Results are mean ± SD (*n* = 3). P values were obtained by comparison with CAST-WT (one-way ANOVA with post hoc Bonferroni’s multiple comparison test, **P < 0.01). Source data are available for this figure: SourceData F6.

**Figure S3.**
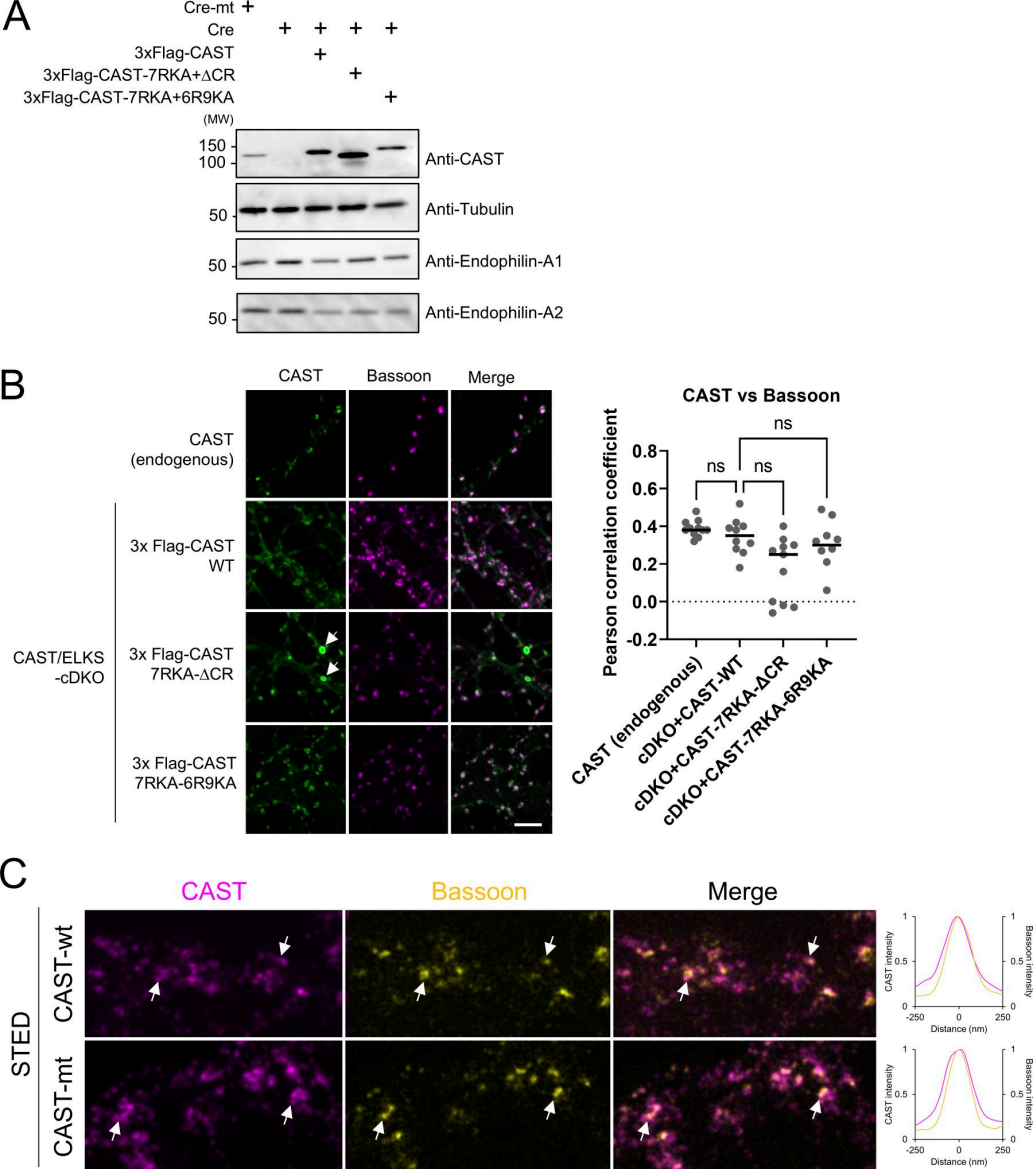
**The interaction with CAST/ELKS with endophilin is required for the stimulation-evoked SV response. (A)** CAST-mutants expression level in mouse hippocampal neurons from CAST-flox/ELKS-flox mouse. CAST and tubulin were detected by the western blot analysis in control (Cre-mutant), CAST/ELKS-cDKO (Cre), CAST/ELKS-cDKO with 3xFlag-CAST-WT (Cre with CaMKII0.3 promoter-derived CAST), with 3xFlag-CAST-7RKA+ΔCR, and with 3xFlag-CAST-7RKA+6R9KA. **(B)** The localization of CAST-mutants in the hippocampal culture neurons. Control, CAST/ELKS-cDKO, CAST/ELKS-cDKO with CAST, or CAST/ELKS-cDKO with CASTmt (CAST-7RKA+ 6R9KA) neurons are stained with CAST and Bassoon. Scale bar: 2 µm. Right dot plots show the correlation co-efficiency between CAST vs Bassoon (*n* = 10 images, each). P values are compared with the cDKO with CAST (one-way ANOVA with post hoc Bonferroni’s multiple comparison test). **(C)** Immunofluorescence images acquired by STED microscopy showing CAST (magenta; WT in the top, mutant [7RKA+6R9KA]) and Bassoon (orange) in CAST/ELKS-cDKO mouse hippocampal neurons expressing either WT or mutant CAST. Arrows indicate sites of co-localization of CAST and Bassoon. Scale bars: 1 µm. The right panels show mean fluorescence intensity profiles from 10 active zones. Source data are available for this figure: SourceData FS3.

Next, we investigated whether CAST/ELKS binding to endophilin-A is essential for the SV release process. To address this, we generated a CAST mutant that does not bind endophilin-A. Specifically, we identified potential endophilin-A–binding regions within the CAST amino acid residues 1–400 fragment (Fig. 4 A and Fig. 6 A) and focused on two conserved regions. The first region, spanning residues 6–42 in mouse CAST, exhibited weak homology with the *Drosophila* CAST ortholog, Bruchpilot (Driller et al., 2019) (Fig. 6 B). This region is rich in basic amino acids (arginine and lysine); therefore, we generated a CAST mutant (CAST-7RKA) by substituting these residues with alanine. The second region, spanning residues 155–212, is highly homologous to Bruchpilot (Wagh et al., 2006) and the *Caenorhabditis elegans* ortholog, Elks-1 (Deken et al., 2005). For this region, we generated two CAST mutants: a deletion mutant (CAST-ΔCR) and one with all arginine and lysine residues replaced with alanine (CAST-6R9KA). To determine their functional effect, we examined co-condensate formation between these mutants and endophilin-A2 in the HEK cells. Individually, CAST-7RKA and CAST-ΔCR had no significant effect on co-condensate formation (Fig. 6 C). However, the combined mutants (CAST-7RKA+ΔCR and CAST-7RKA+6R9KA) showed significantly reduced co-condensate formation efficiency (Fig. 6 C). Binding assays using recombinant proteins further demonstrated that both combined mutants showed <20% of the binding capacity of WT CAST, confirming that the two regions are critical for endophilin-A binding (Fig. 6 D). To investigate the functional effect of these mutants, we expressed WT and mutant CAST proteins fused with 3×Flag tags in hippocampal neurons. Protein expression was confirmed by immunoblotting (Fig. S3 A). Immunostaining with a CAST-specific antibody showed no significant difference between WT and mutant CAST-expressing CAST/ELKS-cDKO neurons in the localization of endogenous CAST at the active zone. However, large aggregates were observed in neurons expressing the CAST-7RKA+ΔCR mutant (Fig. S3 B). To avoid aggregation-related effects, we used the CAST-7RKA+6R9KA mutant, which did not exhibit aggregation. Super-resolution microscopy confirmed that both CAST-WT and CAST-7RKA+6R9KA are located at the active zone (Fig. S3 C).

**Figure 7.**
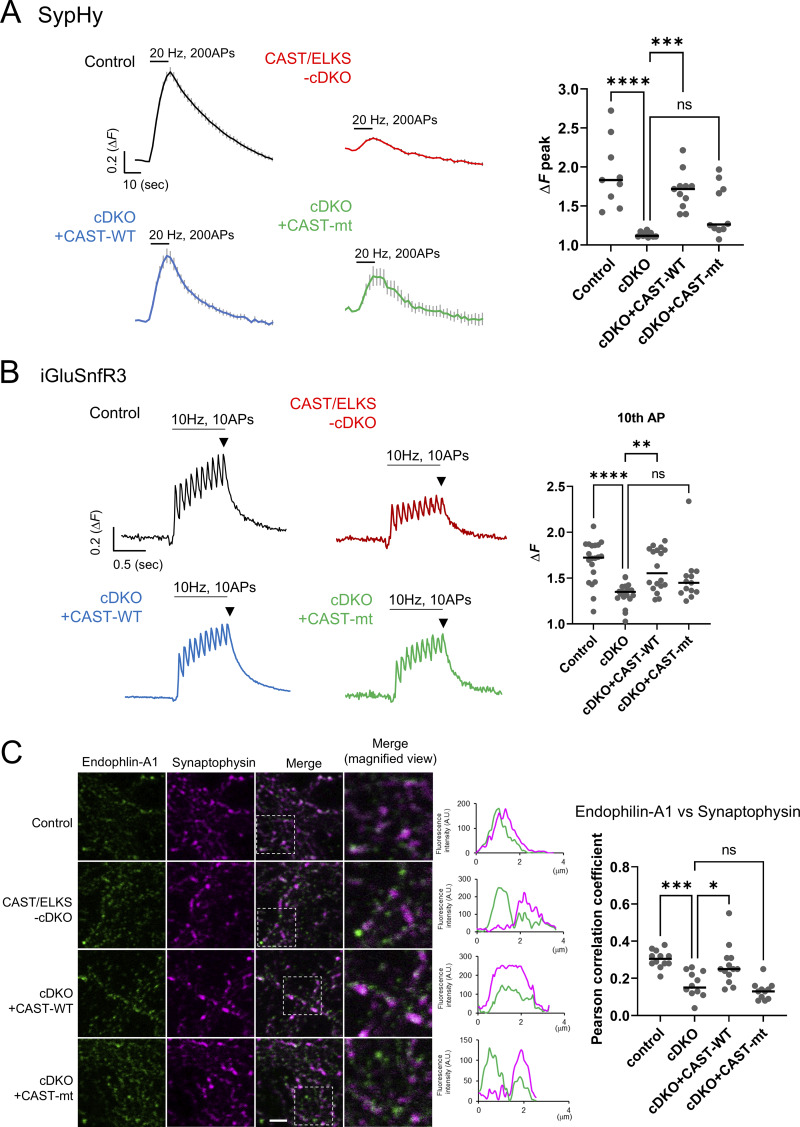
**Interaction of CAST/ELKS and endophilin-A is required for the stimulation-evoked SV response. (A)** Effects of SV release with a CAST mutant. Activity-dependent SypHy fluorescence response in control (black), CAST/ELKS-cDKO (red), CAST/ELKS-cDKO with CAST-WT (blue), or CAST/ELKS-cDKO with CAST-mt (CAST-7RKA+6R9KA) (green) in mouse hippocampal neurons. The traces show average fluorescence of individual SypHy upon 20 Hz stimulation with 200 APs. Right dot plots of the peak of 200 APs. Results are mean ± SEM. *n* = 10 images, each. P values were obtained by comparison with control (one-way ANOVA with post hoc Bonferroni’s multiple comparison test, ***P < 0.001, ****P < 0.0001). **(B)** Activity-dependent iGluSnFR3 fluorescence response in control (black), CAST/ELKS-cDKO (red), CAST/ELKS-cDKO with CAST-WT (blue), or CAST/ELKS-cDKO with CAST-mt (CAST-7RKA+ 6R9KA) (green) in the hippocampal neurons. The left traces show average fluorescence of individual iGluSnFR3 upon 10 Hz, 10 APs stimulation. The arrowheads indicate 10th action potential points. Results are mean ± SEM. *n* = 15 images, each. The right dot plots show 10th AP data. P values are compared with the cDKO-neurons (one-way ANOVA with post hoc Bonferroni’s multiple comparison test, **P < 0.01, ****P < 0.0001). **(C)** Localization of endophilin-A1 in cultured hippocampal neurons. The left panels show control, CAST/ELKS-cDKO, CAST/ELKS-cDKO with CAST-WT, and CAST/ELKS-cDKO with CAST-mt (CAST-7RKA + 6R9KA) neurons stained for endophilin-A1 and synaptophysin. Fluorescence intensity profiles were measured along the dotted lines shown in the images and are presented on the right. The right dot plots show the correlation coefficients between endophilin-A1 and synaptophysin (*n* = 10 images). Scale bar: 10 µm. P values were obtained by comparison with cDKO neurons using one-way ANOVA followed by Bonferroni’s multiple comparison test, *P < 0.05, ***P < 0.001.

We evaluated sustained SV release in CAST/ELKS-cDKO neurons using SypHy fluorescence imaging. Peak SypHy fluorescence, reflecting the capacity to maintain SV release during repetitive stimulation, was significantly reduced in CAST/ELKS-cDKO neurons (Fig. 7 A). This defect was rescued by WT CAST, but not by the CAST-7RKA+6R9KA mutant (Fig. 7 A). Furthermore, neurotransmitter release was examined using iGluSnFR3, which measures glutamate release during action potential stimulation. Fluorescence intensity during the 10th AP was significantly reduced in CAST/ELKS-cDKO neurons and was restored by expression of WT CAST. Expression of the CAST-7RKA+6R9KA mutant resulted in an intermediate level of fluorescence intensity that was not significantly different from either CAST/ELKS-cDKO neurons or WT CAST-expressing neurons (Fig. 7 B). Taken together, these findings suggest that the CAST-7RKA+6R9KA mutant shows an intermediate phenotype that is not statistically distinguishable from either CAST/ELKS-cDKO or WT CAST rescue conditions.

We further investigated whether CAST binding is required for endophilin-A localization at presynaptic terminals. In control neurons, endophilin-A was partially localized to presynaptic terminals. In contrast, endophilin-A localization was significantly reduced in CAST/ELKS-cDKO neurons (Fig. 7 C). Expression of WT CAST partially restored endophilin-A localization, whereas the CAST-7RKA+6R9KA mutant did not rescue this defect (Fig. 7 C). These results indicate that the interaction between CAST and endophilin-A is essential for endophilin-A localization at presynaptic terminals and for supporting activity-dependent SV recycling.

### Endophilin-A1, TRP size, and vesicular pH

**Figure 8.**
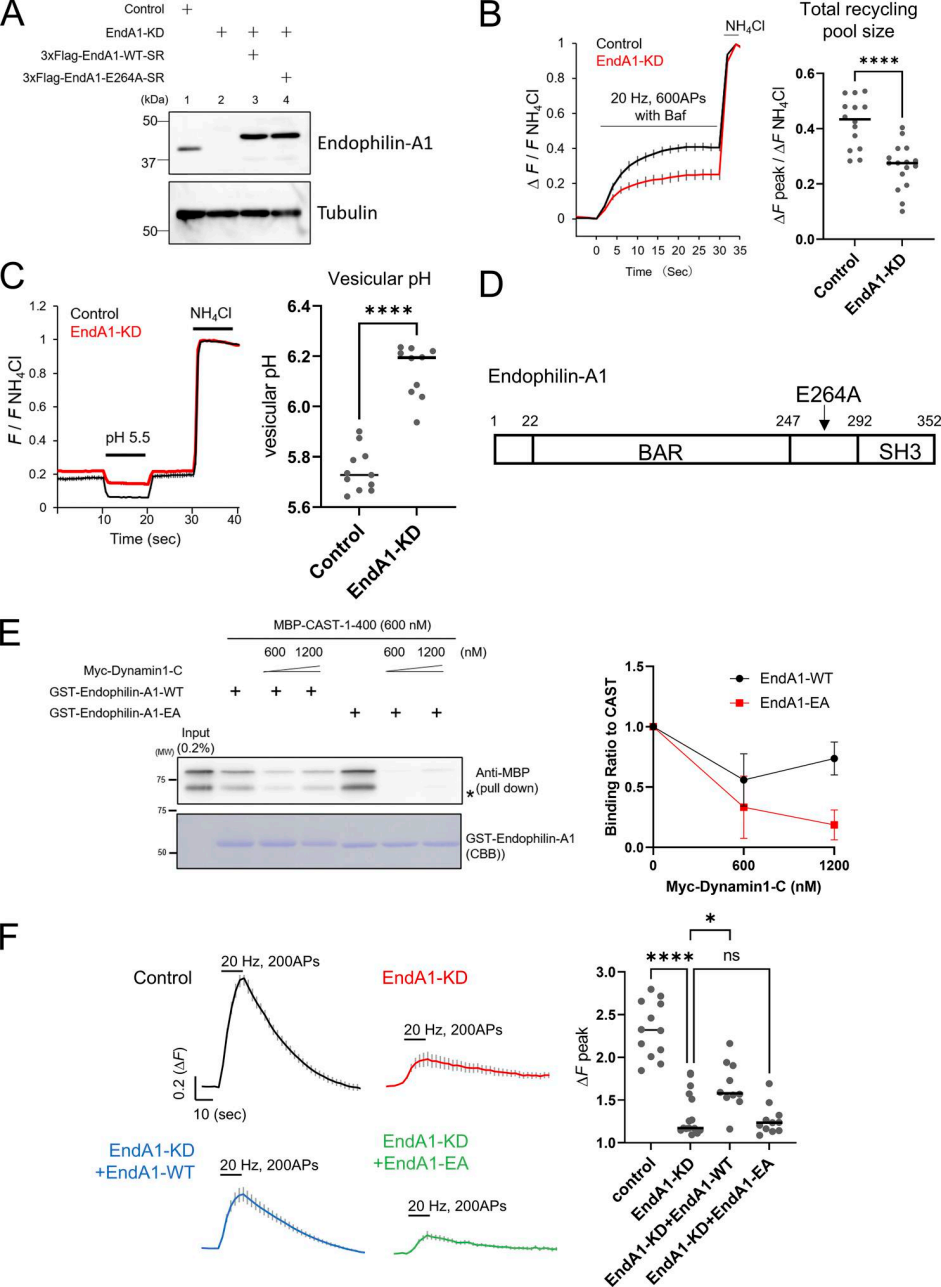
**An endophilin-A linker region mutant exhibits defective binding to CAST and SV responses. (A)** The blot shows EndA1-KD efficiency and endophilin-A1–E264A mutant expression level in rat hippocampal neurons. Endophilin-A1 and tubulin were detected by the western blot analysis in control, EndA1-KD, EndA1-KD rescued with shRNA-resistant 3×Flag-EndA1-WT (3×Flag-EndA1-WT-SR; EndA1 driven by the CaMKII0.3 promoter), and EndA1-KD rescued with shRNA-resistant 3×Flag-EndA1-E264A (3×Flag-EndA1-E264A-SR). **(B)** TRP size in EndA1-KD neurons. Left traces show the TRP size in control (black) or EndA1-KD (red) rat hippocampal cultured neurons. Results are mean ± SEM. *n* = 14 images, each. Right dot plots show the peak of 600 APs. P values were obtained by nonparametric Mann–Whitney test, ****P < 0.0001. **(C)** Vesicular pH of SypHy-bearing vesicles in EndA1-KD rat hippocampal neurons. Left traces show the average for estimating the vesicular pH in control (black) or EndA1-KD (red) neurons. Results are mean ± SEM. *n* = 10 images, each. Right dot plots show quantification of the vesicular pH. P values were obtained by nonparametric Mann–Whitney test, ****P < 0.0001. **(D)** The image shows mouse endophilin-A1–E264A mutant in this study. Glu264 of endophilin-A1 (between the BAR domain and SH3 domain) was changed to Ala. **(E)** Endophilin-A1 mutants altered dynamin-1–dependent binding ability to CAST. Left representative Figures indicates MBP-CAST-1 and dose-dependent Myc-dynamin-1-C (residues 657–867) were precipitated with GST–endophilin-A1 or GST–endophilin-A1-E264A. MBP-CAST-1 and Myc-dynamin-1-C were detected by western blot analysis and GST-endophilin-A1-full was detected by CBB staining. Right line graph shows quantified results of the binding ratio (*n* = 3 independently prepared samples). Results are mean ± SD. **(F)** Effects of SV release in the endophilin-A1 mutant. Activity-dependent SypHy fluorescence response in control (black), EndA1-KD (red), EndA1-KD with endophilin-A1-WT-SR (blue), or EndA1-KD with endophilin-A1-E264A-SR mutant (green) in rat hippocampal cultured neurons. Traces show average fluorescence of individual SypHy upon 20 Hz, 200 APs stimulation. Results are mean ± SEM. *n* = 10 images, each. Right dot plots of the peak of 200 APs. P values were obtained by comparison with EndA1-KD neurons (one-way ANOVA with post hoc Bonferroni’s multiple comparison test, *P < 0.05, ****P < 0.0001). Source data are available for this figure: SourceData F8.

Previous studies have reported that endophilin-A1/A2 DKO neurons exhibit short-term depression (Milosevic et al., 2011), suggesting a role for endophilin-A in regulating TRP size. To investigate this, we examined TRP size in cultured hippocampal neurons by SypHy imaging. Endophilin-A1, the most abundant endophilin-A family protein in the brain (Milosevic et al., 2011), was selectively knocked down using short hairpin RNA in rat hippocampal neurons. Knockdown efficiency was confirmed by immunoblotting, which showed a significant reduction in endophilin-A1 protein levels with no effect on endophilin-A2 expression (Fig. 8 A and Fig. 10 A).

Endophilin-A1 knockdown (EndA1-KD) neurons reduced TRP size to ∼50% of control levels (Fig. 8 B). To further characterize the effect of EndA1-KD, we found that the vesicular pH of SypHy-containing vesicles under basal conditions was significantly increased in EndA1-KD neurons (median: control, pH 5.7; knockdown, pH 6.2) (Fig. 8 C). These observations closely resemble those found in CAST/ELKS-cDKO neurons, suggesting that CAST/ELKS and endophilin-A1 play interconnected roles in regulating TRP size and vesicular acidification.

### Endophilin-A1 mutation alters SV release

To investigate the role of CAST/ELKS binding to endophilin-A in SV release process, we targeted endophilin-A using a mutant-based approach. Previous studies in *Drosophila* have demonstrated that mutation in the spacer region, located between the BAR and SH3 domains of endophilin-A, affected its binding to dynamin-1 (Bademosi et al., 2023). On the basis of this finding, we hypothesized that the E264A mutation in the spacer region would enhance dynamin-1 binding while diminishing the interaction with CAST (Fig. 8 D). To test this hypothesis, we performed pull-down assays using recombinant proteins, including MBP-CAST1-400, endophilin-A1–WT, endophilin-A1–E264A, and Myc-dynamin-1-C. The results showed that in the presence of dynamin-1, binding to the E264A mutant was significantly reduced in a dose-dependent manner, compared with WT endophilin-A1 (Fig. 8 E). These findings indicate that the E264A mutation enhances dynamin-1 affinity while impairing the ability of endophilin-A to interact with CAST.

**Figure S4.**
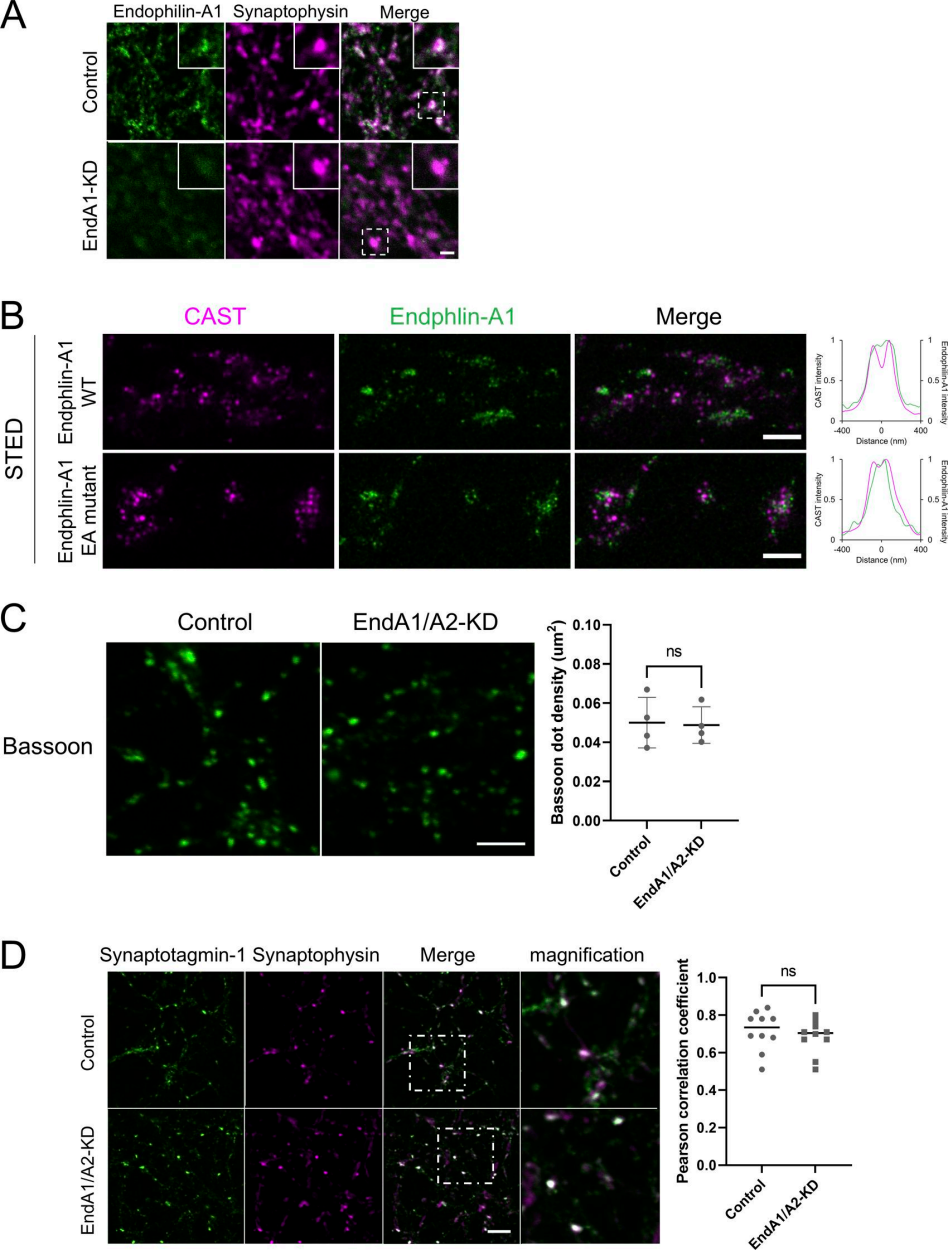
**Localization of the endophilin-A1 E264A variant and the roles of endophilin-A1 and endophilin-A2. (A and B)** Cultured rat hippocampal neurons (control or EndA1-KD) were stained for endophilin-A1. The specificity of the endophilin-A1 antibody was confirmed by a marked reduction in fluorescence signal following EndA1-KD. Scale bar: 2 µm (B) Immunofluorescence images acquired by STED microscopy showing CAST (magenta) and endophilin-A1 (green; WT in the top, E264A mutant in the bottom) in EndA1-KD rat hippocampal neurons expressing either WT or mutant endophilin-A1. Scale bars: 1 µm. The right panels show mean fluorescence intensity profiles from 10 active zones. **(C)** The left panel shows immunofluorescence images of Bassoon (green) in control and EndA1/A2-KD rat hippocampal neurons. Scale bar: 2 µm. The dot plots on the right show the area of Bassoon puncta in control and EndA1/A2-KD neurons (∼10–50 puncta were analyzed per image from 20 images per group, and the mean was calculated; the experiment was repeated four times using independent cultures). Error bars indicate SD (*n* = 4). P values were obtained by nonparametric Mann–Whitney test. **(D)** Rat hippocampal cultured neurons (control or EndA1/A2-KD) were stained for synaptotagmin-1 and synaptophysin. The dot plots on the right show the correlation coefficients between synaptotagmin-1 and synaptophysin (*n* = 10 images per group). Scale bar: 2 µm. P values were obtained by nonparametric Mann–Whitney test.

Next, we examined whether the E264A mutation affected SV release in EndA1-KD neurons. We knocked down endophilin-A1 and/or endophilin-A2 and expressed either WT or the E264A mutant and examined their localization using STED microscopy, finding no detectable differences between them (Fig. S4, A and B). The E264A mutant failed to rescue the reduction in SypHy fluorescence peak observed in EndA1-KD neurons, whereas WT endophilin-A1 successfully restored this defect (Fig. 8 F). These findings demonstrate that the interaction between endophilin-A and CAST is crucial for SV release. Furthermore, the inability of the E264A mutant to compensate for this defect highlights the importance of CAST binding in this process.

### Regulation of CLC presynaptic distribution by CAST/ELKS–endophilin-A

**Figure 9.**
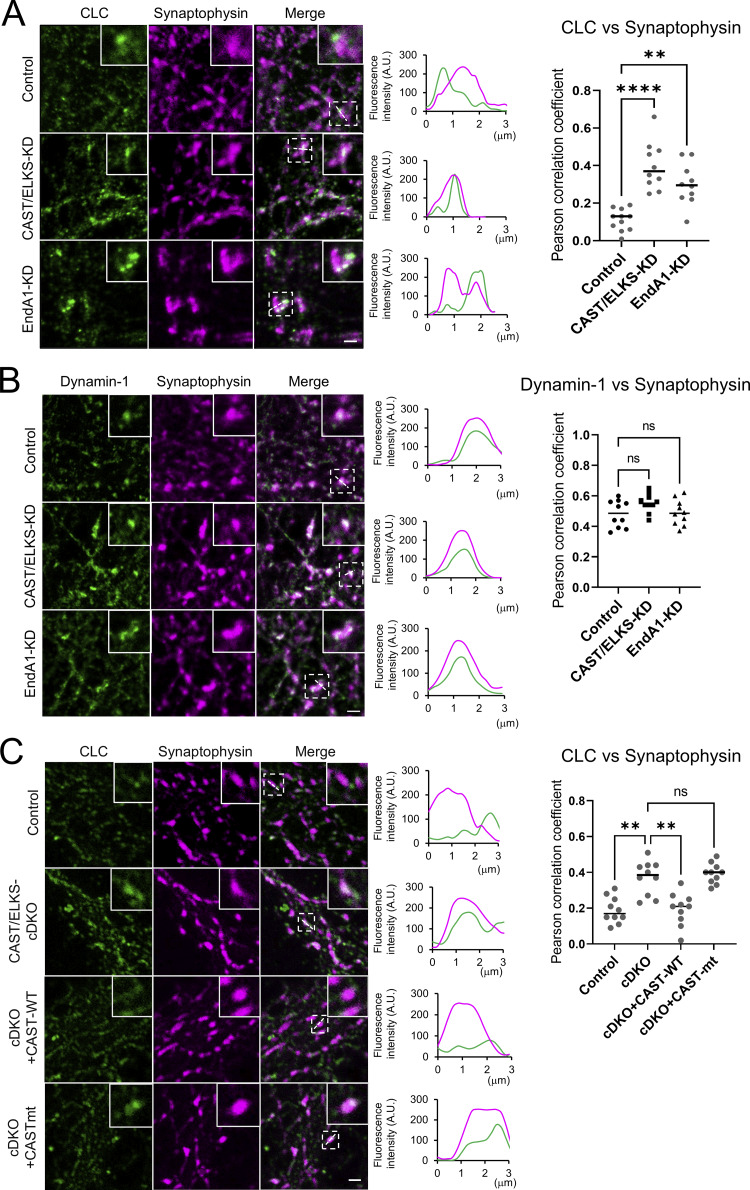
**Binding of CAST to endophilin-A is required for the presynaptic localization of CLC. (A and B)** Localization of endosomal proteins in CAST/ELKS or EndA1-KD neurons. Control, CAST/ELKS-KD, or EndA1-KD rat hippocampal cultured neurons stained with CLC (A) or dynamin-1 (B) and synaptophysin. Fluorescence intensity profiles were measured along the dotted lines shown in the images and are presented on the right. Right dot plots show the correlation co-efficiency between CLC vs. synaptophysin (A) and dynamin-1 vs. synaptophysin (B) (*n* = 10 images, each). P values were obtained by comparison with control neurons. Bar = 2 μm (one-way ANOVA with post hoc Bonferroni’s multiple comparison test, **P < 0.01, ****P < 0.0001). **(C)** Localization of CLC in CAST/ELKS-cDKO neurons. control, CAST/ELKS-cDKO, CAST/ELKS-cDKO with CAST-WT, or CAST/ELKS-cDKO with CAST-mt (CAST-7RKA+6R9KA) in mouse hippocampal cultured neurons stained with CLC and synaptophysin. Right dot plots show the correlation co-efficiency between CLC and synaptophysin (*n* = 10 images, each). P values were obtained by comparison with cDKO neurons (one-way ANOVA with post hoc Bonferroni’s multiple comparison test, **P < 0.01). Scale bars: 2 μm.

**Figure. S5.**
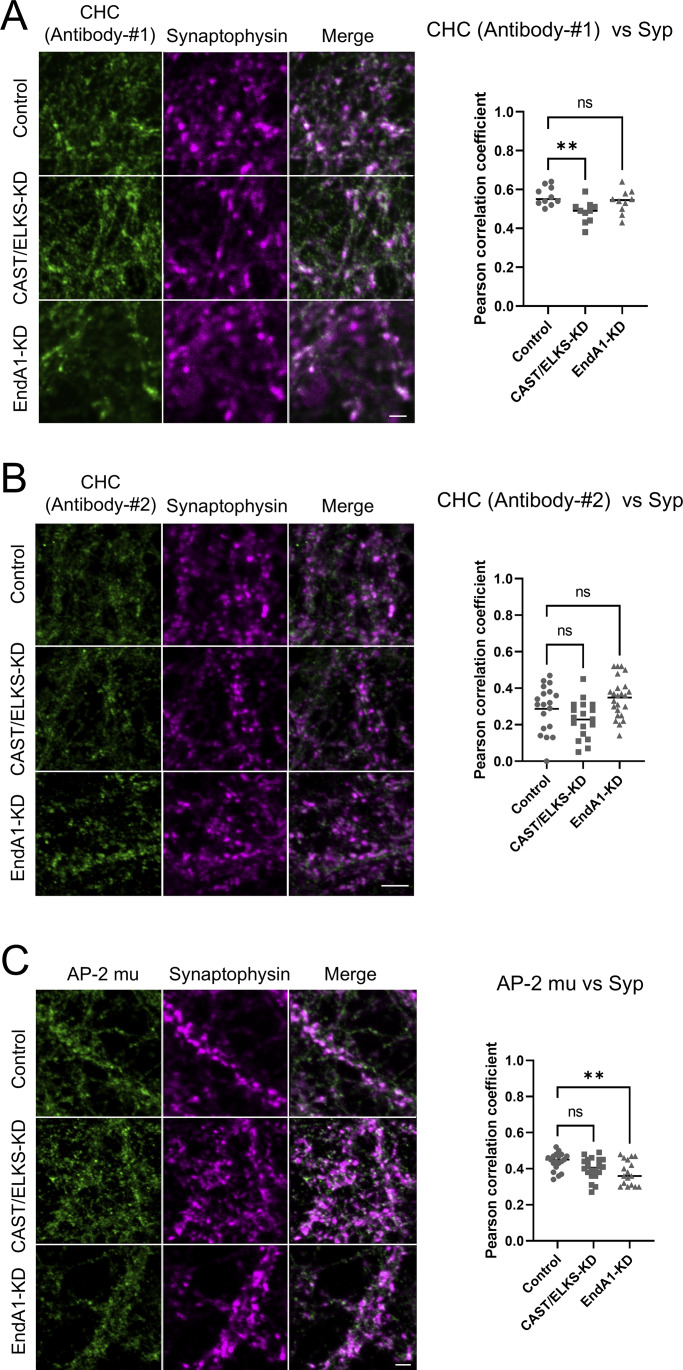
**Localization of CHC and AP-2 in CAST/ELKS- or EndA1-KD neurons. (A–C)** Rat hippocampal neurons in culture (control, CAST/ELKS-KD, or EndA1-KD) were immunostained for CHC (A and B; anti-CHC antibodies: A, CHC antibody-#1 [catalog no. ab21679; Abcam]; B, CHC antibody-#2 [catalog no. 2410; Cell Signaling]), AP-2 μ (C), and synaptophysin. Dot plots on the right show correlation coefficients between CHC and synaptophysin (A: *n* = 10 images per group; B: control, *n* = 19; CAST/ELKS-KD, *n* = 18; EndA1-KD, *n* = 24), as well as between AP-2 and synaptophysin (C: control, *n* = 19; CAST/ELKS-KD, *n* = 20; EndA1-KD, *n* = 17). P values were calculated relative to control neurons using the nonparametric Mann–Whitney test, **P < 0.01. Scale bars, 2 µm.

Previous study described that during repetitive stimulation, endophilin-A disperses from presynaptic terminals and subsequently returns after the stimulation (Yoshida et al., 2023). Conversely, CLCs accumulate at presynaptic terminals during neuronal activity and disperse after stimulation (Loerke et al., 2005; Mueller et al., 2012). Thus, endophilin-A and CLCs exhibit opposite activity-dependent redistribution patterns at presynaptic sites. Given that CAST/ELKS deletion reduces the presynaptic abundance of endophilin-A (Fig. 7 C), we hypothesized that disruption of the CAST–endophilin-A pathway might shift the distribution of CLCs toward presynaptic retention or accumulation. To test this possibility, we examined the localization of CLCs together with other endocytic proteins in CAST/ELKS-cDKO and EndA1-KD neurons. We performed immunostaining for CLC, CHC, dynamin-1, AP-2, and synaptophysin as a presynaptic terminal marker. In control neurons, the correlation coefficient between CLC and synaptophysin was low (median <0.20), suggesting that CLC is not preferentially localized at presynaptic terminals (Fig. 9 A, top panels). However, in CAST/ELKS-cDKO and EndA1-KD neurons, the correlation coefficient was significantly higher, suggesting altered retention of CLC at presynaptic terminals (Fig. 9 B, middle and bottom panels). Notably, the correlation coefficient between synaptophysin and dynamin-1, CHC, or AP-2 was consistently higher than that for CLC and remained unchanged following knockdown of CAST/ELKS or endophilin-A1 (Fig. 9 B and Fig. S5). This suggests that CAST/ELKS specifically regulate the CLC localization at presynaptic sites, rather than dynamin-1, CHC, or AP-2. To further confirm the role of CAST–endophilin-A binding in CLC localization at presynaptic sites, we expressed WT and mutant CAST in CAST/ELKS-cDKO neurons. Expression of WT CAST successfully rescued the colocalization defect between CLC and synaptophysin, whereas mutant CAST failed to restore normal dynamics (Fig. 9 C). These findings show that the interaction between CAST and endophilin-A is crucial for CLC localization at presynaptic sites.

### Endophilin-A stabilizes active zone proteins

**Figure 10.**
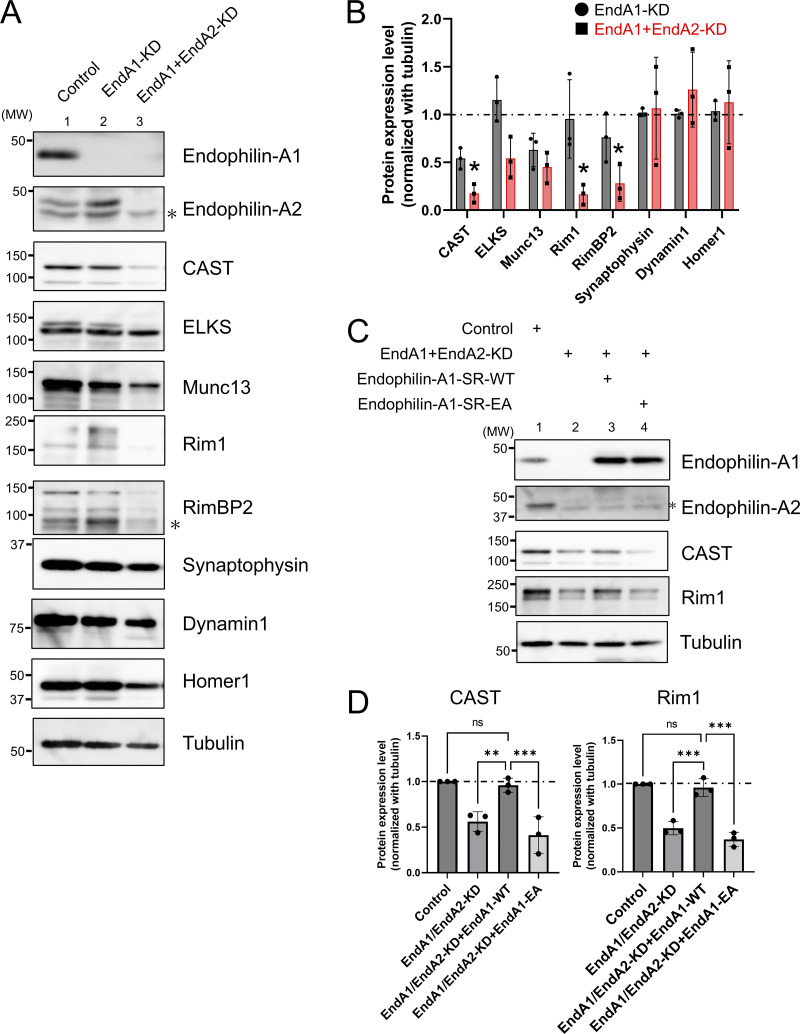
**Endophilin-A is necessary for stabilizing active zone proteins. (A)** Protein expression levels of synapse proteins in endophilin-A-knockdown neurons. Endophilin-A1, endophilin-A2, CAST, ELKS, Munc13, Rim1, RimBP2, synaptophysin, dynamin-1, Homer1, and tubulin were detected by western blot analysis in control, EndA1-KD, or EndA1-KD and Endophilin-A2-KD rat hippocampal neurons. **(B)** Quantification of the results of (A). Protein expressions in EndA1-KD, or EndA1-KD and Endophilin-A2-KD neurons were normalized with tubulin and further normalized by control neurons. Results are mean ± SD. *n* = 3 independently prepared samples. P values were obtained by comparison with control neurons (nonparametric Mann–Whitney test, *P < 0.05). **(C)** Effect on active zone protein expression levels in the endophilin-A1–E264A mutant. Endophilin-A1, endophilin-A2, CAST, Rim1, and tubulin were detected by western blot analysis in control, EndA1-KD and endophilin-A2-KD, EndA1-KD and endophilin-A2-KD with endophilin-A1-WT, or EndA1-KD and endophilin-A2-KD with endophilin-A1–E264A in rat hippocampal neurons. **(D)** Quantification of the results of (C). CAST and Rim1 expression levels were normalized with Tubulin and further normalized by control neurons. Results are mean ± SD. *n* = 3 independently prepared samples. P values were obtained by comparison with EndA1-KD and endophilin-A2-KD (one-way ANOVA with post hoc Bonferroni’s multiple comparison test, **P < 0.01, ***P < 0.001). Source data are available for this figure: SourceData F10.

We investigated the effect of endophilin-A on active zone proteins by performing knockdown of endophilin-A1 and endophilin-A2. Previous analyses using antibodies that recognize all endophilin-A isoforms have shown that endophilin-A1 is the predominant isoform in the brain, while endophilin-A2 is only present in small amounts (Milosevic et al., 2011). In addition, studies in inner hair cells have shown that calcium influx is already reduced by a single loss of endophilin-A1, whereas a reduction in SV number near the ribbon/active zone is observed only when both endophilin-A1 and A2 are simultaneously disrupted (Kroll et al., 2019). Therefore, we conducted our investigations under conditions in which only endophilin-A1 was knocked down, as well as conditions in which both endophilin-A1 and endophilin-A2 were knocked down. Knock down of endophilin-A1, the most abundant isoform in neurons, did not significantly alter the expression levels of active zone proteins. However, simultaneous knockdown of both endophilin-A1 and endophilin-A2 resulted in a significant reduction in CAST, Rim1, and RimBP2 levels (Fig. 10, A and B), while the expression levels of ELKS and Munc13 did not differ significantly. Additionally, expression levels of other presynaptic and postsynaptic proteins (such as dynamin-1, synaptophysin, and Homer1) showed no significant changes. These findings suggest that both endophilin-A1 and endophilin-A2 are essential for the maintenance of specific active zone proteins, particularly CAST and Rim1. Furthermore, the reduced expression levels of CAST and Rim1 in endophilin-A1/A2-double-knockdown neurons were restored by expressing WT endophilin-A1, but not the endophilin-A1–E264A mutant (Fig. 10, C and D). On the other hand, no significant changes in Bassoon puncta size (Fig. S4 D) or synaptotagmin-1 (Fig. S4 E) fluorescence levels were observed following the knockdown of endophilin-A1 and endophilin-A2. These results indicate that the interaction between endophilin-A and CAST is crucial for maintaining the expression levels of key active zone proteins.

## Discussion

In this study, we showed that CAST/ELKS regulate the TRP size as well as the RRP size of SVs. Through comprehensive screening in the HEK cells, we identified endophilin-A as a CAST/ELKS-associated protein. Mutations in CAST that prevent binding to endophilin-A led to decreased SV release, presynaptic localization of endophilin-A, and altered presynaptic distribution of CLC. Moreover, the endophilin-A1–E264A mutation, hindering dynamin-1–dependent binding to CAST, interfered with SV release and influenced the stability of active zone proteins, including CAST and Rim1. These findings collectively support the conclusion that CAST/ELKS promote the presynaptic function of endophilin-A through a physical interaction.

CAST/ELKS have previously been implicated in regulating RRP size and synaptic release probability through mechanisms such as calcium influx at presynaptic terminals. Our study clarifies this further by showing that CAST/ELKS also maintains TRP size by modulating the function of endophilin-A. This suggests that CAST/ELKS not only govern the response of some SVs near the presynaptic membrane but also control the dynamics of a broad population SVs within the presynaptic terminal through regulation of endophilin-A. While several studies have proposed that RRP size is determined by vesicles bound to the active zone, other studies using electron microscopy analyses suggest that the number of docked SVs does not always correlate with RRP size, highlighting an ongoing debate (Kaeser and Regehr, 2017). We propose that TRP size may be a key factor influencing RRP size. In other words, the proportion of functional SVs throughout the presynaptic terminal can contribute to determining RRP size rather than just those in contact with the active zone.

Our fluorescent protein–based observations of SV dynamics further support the idea that both CAST/ELKS and endophilin-A play critical roles in maintaining TRP size. Although other proteins such as CDK5 and synapsin have been shown to regulate TRP size (Kim and Ryan, 2010), the precise mechanism remains unclear. Based on our observation that CLC shows altered accumulation at presynaptic sites following knockdown of CAST/ELKS and endophilin-A, we propose that the dynamic redistribution of some endocytic proteins contributes to SV recycling and helps maintain the size of the SV pool. Recent studies have suggested that endophilin-A, in addition to its established role in endocytosis, contributes to maintaining the size of the SV pool through interactions with intersectin-1 (Ogunmowo et al., 2025). Other studies have proposed that endophilin-A undergoes LLPS with synapsin (Yoshida et al., 2023). Taken together with our findings, these observations support a model in which endophilin-A, recruited to functional presynaptic regions via CAST/ELKS, organizes the overall SV recycling process. In this model, endophilin-A contributes to multiple steps involved in the retrieval of SV membrane lipids and proteins, as well as to the regeneration of functional SVs after endocytosis and maintaining the size of both the RRP and the broader recycling pool. Future studies should examine how CAST/ELKS contributes to interactions among proteins involved in the functional SV pool.

Our investigation into the physical interactions among CAST, endophilin-A, and dynamin-1 showed that dynamin-1 negatively regulates the binding of CAST to endophilin-A (Fig. 4, C and D). In addition, we found that the E264A mutant, which carries a substitution in the spacer region of endophilin-A1, shows a dynamin-1–dependent decrease in its interaction with CAST compared with WT endophilin-A1 (Fig. 8 E). Previous studies reported that a mutant form of *Drosophila* endophilin-A, in which D265—the residue corresponding to E264—is replaced with alanine (D265A mutant), is predicted to have reduced flexibility in the spacer region (Bademosi et al., 2023). Taken together, these results suggest that the interaction between dynamin-1 and endophilin-A regulates CAST–endophilin-A binding and that the flexibility of the spacer region plays a key role in this regulation. We did not detect a ternary complex containing CAST, dynamin-1, and endophilin-A under our experimental conditions, suggesting that these interactions are mutually exclusive. Consistently, SV recycling measured with SypHy in CAST/ELKS cDKO neurons showed no difference in the rate of fluorescence decay after stimulation compared with WT neurons (Fig. 2 A). These results further support the conclusion that CAST/ELKS does not appear to directly contribute to dynamin-dependent processes. Notably, the SH3 domain of endophilin-A interacts with several presynaptic proteins, including VGLUT1, synaptojanin-1, and intersectin-1 (Ringstad et al., 1997; Voglmaier et al., 2006; Pechstein et al., 2015). Future studies should examine the endophilin-A1 E264A mutant, which shows reduced CAST/ELKS binding in a dynamin-1–dependent manner, and determine whether SH3-binding proteins such as intersectin-1 are excluded from or incorporated into the CAST/ELKS–endophilin-A1 complex. It will also be important to assess how these interactions influence endophilin-A–mediated curvature sensing and clathrin dynamics during SV recycling.

In the present study, we found that knockdown of CAST/ELKS or endophilin-A1 leads to an association of CLC immunoreactivity with presynaptic marker, whereas the immunoreactivity of CHC and the adaptor protein AP-2 remains largely unchanged. Because the changes detected in our analysis were more pronounced for CLC than for CHC or AP-2, the observed changes in CLC localization may reflect alterations in its redistribution between presynaptic and extrasynaptic regions. Consistent with this idea, CLC has been shown to accumulate at presynaptic sites during neuronal activity and subsequently disperse, and extrasynaptic CLC puncta have also been reported (Mueller et al., 2004; Loerke et al., 2005). Taken together, these findings suggest that the altered presynaptic CLC signal reflects changes in the spatial regulation or redistribution of CLC, rather than a uniform change in clathrin complex behavior. Further studies will be required to clarify how CAST/ELKS and endophilin-A1 contribute to the activity-dependent positioning and trafficking of CLC within presynaptic terminals.

Recently, multiple types of presynaptic protein condensates—such as synapsin condensates (Milovanovic et al., 2018), active zone condensates (McDonald et al., 2020; Wu et al., 2019; Qiu et al., 2024), and endocytic protein condensates (Imoto et al., 2022; Mondal et al., 2022; Yoshida et al., 2023). Because SV recycling is a continuous and coordinated process, interactions among these condensates are likely to be important; however, the underlying molecular mechanisms remain largely unclear (Choi et al., 2024). In this study, we found that CAST/ELKS and endophilin-A form co-condensates in the HEK cells. We also observed that CAST/ELKS, an active zone protein, regulates the localization of endophilin-A, which plays a role in endocytosis and in maintaining the size of the SV pool. Conversely, endophilin-A influenced the protein levels of the active zone proteins CAST/ELKS, Rim1, and Rimbp2. These findings suggest that, within the presynaptic structure, LLPS-mediated interactions occur not only between proteins in the same region but also transiently between different regions. Such interactions may contribute to the integration of SV recycling, which is involved in the maintenance of the SV pool, as well as to the stabilization of presynaptic proteins. Future studies should examine whether LLPS occurs more broadly between CAST/ELKS and other presynaptic proteins, which may provide further insight into the molecular organization of synaptic terminals. In addition, experiments using neurons will be required to clarify how functional interactions among SV proteins, active zone proteins, and endocytic proteins contribute to the maintenance of the SV pool in the brain.

## Materials and methods

### Mouse strains

The use of animals was approved by the Institutional Committee for the Care and Use of Experimental Animals at the University of Yamanashi. Mice were housed in a 12/12 h light/dark cycle with food and water available *ad libitum*. *CAST/ELKS* double-floxed mice (Hagiwara et al., 2018) were generated by cross-breeding *CAST*^*flox/flox*^ mice with *ELKS*^*flox/flox*^ mice.

### Antibodies and plasmids

Lentiviral or AAV packaging plasmids, CMV- or CAG-promoter–based mammalian expression plasmids, bacterial expression plasmids, primers and oligonucleotides used in plasmid construction, and antibodies used for immunoblotting and immunostaining are listed in Tables S1 and S2.

### Primary hippocampal culture, lentivirus, and AAV production

Primary hippocampal cultures were prepared from *CAST/ELKS* double-floxed mouse brains (postnatal day 1) or Wistar rat brains (embryonic day 18), as described previously, with slight modifications (Hamada et al., 2021). Briefly, hippocampi were dissected and dissociated by papain at 37°C. After digestion, hippocampal cells were plated at a cell density of 20,000–30,000 cells/cm^2^ in neurobasal medium containing 10% FBS on poly-D-lysine–coated coverslips in either 24-well plates or 4-well dishes (Thermo Fisher Scientific). Cells were incubated with 5% CO_2_ at 37°C. The medium was exchanged after 4–24 h to neurobasal medium containing B-27 supplement (Gibco) and GlutaMAX (Gibco). At 3−5 days *in vitro* (DIV), 2.5 μM of 1-β-D-arabinofuranosylcytosine (Sigma–Aldrich) was added to inhibit the growth of glial cells. For examining CAST/ELKS function, neurons from *CAST/ELKS* double-floxed mice were transduced with AAV expressing Cre or Cre-mt at 3–5 DIV. Other AAV or lentiviruses were transduced at 7–10 DIV. Neurons were subjected to experiments at 17–22 DIV. Lentivirus or AAV production was performed as described previously (Mori et al., 2021; Hamada et al., 2021).

### Live imaging

Fluorescence imaging was conducted at RT (∼23°C) using an inverted microscope (IX73, Olympus) equipped with a custom-made imaging chamber, which featured a 14-mm glass-bottomed dish placed on a movable stage and with a 100× oil immersion objective. The excitation light was obtained from X-cite X-LED1 (Excelitas Technologies Corp.). Neural cells were cultured on glass coverslips (Matsunami) and placed in 2 ml of standard extracellular solution containing (in mM): 140 NaCl, 2.4 KCl, 10 HEPES, 10 glucose, 2 CaCl_2_, 1 MgCl_2_, and 0.02 cyanquixaline (pH 7.4) on 14-mm diameter glass-bottomed dishes (BCFC14-10N; Bio Medical Science Inc.). Field stimulation at various frequencies (from 10 or 20 Hz; indicated in each figure) was delivered via bipolar platinum electrodes with 1 ms constant voltage pulses (5 V) controlled by Master-9 with Iso-flex (AMPI). To measure the kinetics of exocytosis and TRP size, neurons were treated for 1 min with standard extracellular solution containing 0.5 μM bafilomycin A1 (Sigma–Aldrich). The solution was then changed to standard extracellular solution alone for stimulation with 600 action potentials at 20 Hz, with NH_4_Cl extracellular solution containing (in mM): 50 NH_4_Cl, 90 NaCl, 2.4 KCl, 10 HEPES, 10 glucose, 2 CaCl_2_, and 1 MgCl_2_ (pH 7.4) applied directly onto the area of interest using a fast flow exchange microperfusion device and a bulb controller controlled by Master-9. To measure the RRP size, neurons were treated for 0.25 s with standard extracellular solution containing 0.25 M sucrose (Nacalai) applied by microperfusion as already described. To measure surface expression levels of SypHy and vesicular pH, neurons were placed in calcium-reduced extracellular solution containing (in mM): 140 NaCl, 2.4 KCl, 10 HEPES, 10 glucose, 0.2 CaCl_2_, and 2.8 MgCl_2_ (pH 7.4) at 30 s, applied with low pH solution (in mM): 140 NaCl, 2.4 KCl, 10 HEPES, 10 glucose, 0.2 CaCl_2_, and 2.8 MgCl_2_ (pH 5.5) at 10 s, and applied again with calcium-reduced extracellular solution at 10 s and then NH_4_Cl extracellular solution containing (in mM): 50 NH_4_Cl, 90 NaCl, 2.4 KCl, 10 HEPES, 10 glucose, 0.2 CaCl_2_, and 2.8 MgCl_2_ (pH 7.4) at 10 s. Fluorescence images (960 × 600 pixels) were acquired with an ORCA-SPARK sCMOS camera (Hamamatsu Photonics) in time-lapse mode at either 1 Hz (for SypHy imaging at the stimulation condition), 2 Hz (for SypHy imaging measurement of the surface expression level and vesicular pH), 67 Hz (for Syn-GCaMP8s or iGluSnFR3 imaging at the stimulation condition), or 0.5 Hz (for RRP measurement by iGluSnFR3), control of MetaMorph software (Molecular Devices). Fluorescence of SypHy, Syn-GCaMP8s, or iGluSnFR3 was imaged with 470/22 nm excitation and 514/30 nm emission filters.

### Live image data analysis

For calcium imaging with Syn-GCaMP8s, glutamate imaging with iGluSnFR3, and SV release events with SypHy, acquired fluorescence images were automatically analyzed using the SynActJ plugin in Fiji according to the instruction manual (Schmied et al., 2021). Regarding the fluorescence values obtained from one image, the fluorescence value at the time before stimulation was defined as *F*_0_, and the respective fluorescence values (*F*) were divided by *F*_0_ to produce the value (Δ*F*). For each average trace, τ_exo_ was calculated using GraphPad Prism software (GraphPad Software, www.graphpad.com). In the case of monitoring RRP size, TRP size, rise kinetics, surface expression level, and SV pH, acquired fluorescence images were manually analyzed using MetaMorph software, as described previously (Mori et al., 2021). Briefly, active synapses were identified manually by changes in live measurements of fluorescence. Circular regions of interest (ROIs; 2.26 μm diameter and 4 μm^2^ area) were positioned manually at the center of the highlighted fluorescence spots, and an average trace from one image (containing 10–15 active boutons) was collected, and taken as *n* = 1.

### Condensate formation analysis

HEK293T cells were cultured on 14-mm diameter glass-bottomed dishes (FC14-10N, FPI). After 24 h, the cells were transfected with each EGFP-tagged or mCherry-tagged DNA. Fluorescence images were obtained using a laser scanning confocal microscope (FV1000, Olympus) equipped with a 100× (1.35 NA) oil immersion objective. Obtained images were quantified using FV1000 image software (Olympus). Briefly, the region encompassing the extracellular, cytoplasmic, and condensate areas was connected by a 20-μm long straight line, and fluorescence values along this line were measured using a line scan. Average fluorescence intensities of the extracellular, cytoplasmic, and condensate regions were calculated. The extracellular fluorescence value was used as the background, and background-subtracted values for the cytoplasmic and condensate regions were calculated and designated as *a* and *b*, respectively. The ratio (*b/a*), obtained by dividing *b* by *a*, was determined from 10 cells per sample.

### FRAP analysis

HEK293T cells were transfected with expression plasmids encoding the indicated fluorescence-tagged protein and then subcultured on collagen-coated glass-bottomed dish for 24 h. Prior to imaging, the culture medium was replaced with Hank’s balanced salt solution supplemented with 0.1% FBS. A FLUOVIEW FV1200 imaging system (Olympus) was used to capture time-lapse images with a 60× 1.35 NA oil immersion objective. Images were taken at 2.278-s intervals per frame, after 100 ms of photobleaching with a 405 nm laser at 90% of its maximum power in a simultaneous imaging mode, using appropriate digital zoom. Captured images were analyzed using Fiji.

### ALFA-tag protein precipitation assay

The ALFA-tag protein precipitation assay for detecting protein–protein interactions in cellular conditions was used as follows. HEK293T cells were transfected with DNA for each expression plasmid: ALFA-mCherry–tagged proteins and EGFP-tagged proteins. The HEK cell lysates were prepared in lysate buffer containing 1% Triton X-100 in PBS supplemented with Complete, EDTA-free Protease Inhibitor-Cocktail (Roche). For ALFA-tag precipitation, 40 μL of glutathione-Sepharose beads (50% slurry; Cytiva) were incubated with 5 mg of recombinant ALFA-nanobody-tagged GST protein at 30 min in lysate buffer, then the beads were washed twice with lysate buffer. Glutathione–Sepharose beads coupled with ALFA-nanobody GST were incubated with the HEK cell lysate for 30 min, washed three times in lysate buffer, and then eluted in SDS sample buffer. ALFA-mCherry–tagged proteins and EGFP-tagged proteins were detected by immunoblot analysis.

### GST-pull-down assays

GST-pull-down assay was performed to determine the content of wild MBP-CAST-1-400 WT or mutant proteins using GST–endophilin-A proteins. For the GST-pull-down assay, 2 μg of GST-fused endophilin-A protein-coupled glutathione-Sepharose beads (Cytiva) was incubated with 30 μg of MBP-CAST-1 protein, and Myc-dynamin1-C (residues 657–868) was added to the specified molar concentration at 4°C for 1 h in binding buffer (25 mM Tris-HCl, pH 7.5, 150 mM NaCl, 1% Triton X-100, 1 mM DTT, and 0.5 mM EDTA). After incubation, reaction mixtures were extensively washed with the same buffer. Proteins were then eluted in Laemmli sample buffer by boiling, resolved via SDS-PAGE, and detected via western blotting or Coomassie blue staining.

### Western blot analysis

Cells were washed with PBS and then proteins extracted with SDS sample buffer or PBS containing 1% Triton X-100 with complete and EDTA-free Protease Inhibitor-Cocktail (Roche). The samples were separated by SDS-PAGE and transferred to a polyvinylidene fluoride membrane. The membrane was blocked with 5% skimmed milk in TBS with Tween 20 (TBST) for 0.5–1  h, and then incubated with primary antibodies for 1  h at RT or overnight at 4°C. This was followed by incubation with horseradish peroxidase-conjugated secondary antibody for 1 h, with TBST washes between incubations. Chemical luminescence was detected using Immobilon Forte (Millipore) and a LAS4000 mini imager (GE Healthcare Life Sciences). The asterisks in each figure indicate nonspecific bands. Band intensities were measured using Fiji, the expanded version of ImageJ.

### Immunofluorescence

Cultured neurons were fixed with 4% (wt/vol) paraformaldehyde (Wako) for 10 min at RT. After washing with PBS, cells were permeabilized with PBS containing 0.1% Triton X-100 for 20 min at RT and incubated with PBS containing 10% (vol/vol) FBS for 30 min at RT. Cells were incubated with primary antibodies for 1 h at RT or at 4°C overnight. Cells were rinsed 3× with PBS containing 0.05% of Tween-20 and further incubated with Alexa-conjugated IgG (Thermo Fisher Scientific) for 30 min at RT in PBS containing 0.05% of Tween-20. Fluorescence images were obtained with a laser scanning confocal microscope (FV1000, Olympus) equipped with a 100× (1.35 NA) oil immersion objective. Pearson’s colocalization score was obtained through the ImageJ Coloc2 plugin.

### STED imaging

For two color STED imaging, specimens were immunostained with secondary antibodies labeled with Alexa Fluor 594 NHS Ester (Thermo Fisher Scientific) and STAR635P NHS Ester (Abberior). Postfixed specimens were mounted in ProLong Glass Antifade Mountant (Thermo Fisher Scientific) and sandwiched with another small coverslip. STED imaging was performed using a Leica TCS SP8 STED 3× microscope equipped with a pulsed white light laser, a continuous 592 nm STED laser (for alignment), a pulsed 775 nm STED laser, two HyD-SMD detectors, and a 100× oil immersion objective (NA = 1.40). Excitation laser wavelengths were set to 561 nm for Alexa Fluor 594 and 633 nm for STAR635P. The power of all excitation lasers was set to 25% of the maximum. The 775 nm STED depletion laser power was set to 75–100% of its maximum power (∼300 mW), and delay time was set to 0−300 ps. Alignment of the STED depletion laser was performed every 30 min. STED images of 1024 × 1024-pixel resolution were scanned at 400 Hz with 8× line accumulation. The optical zoom factor was set to 11.36, and the resulting image pixel size was 10.0 nm. Detectors were configured to counting mode with a gating from 0.5 to 6.5 ns. For comparison, confocal images of the same field of view (with 775 nm STED laser power 0%) were scanned prior to obtaining STED images. All images were acquired using Leica LAS-X software and stored as 16-bit images.

To calculate the distances between endophilin-A1 puncta and their nearest active zones, STED images of endophilin-A1 and CAST were processed using unsharp masking. Specifically, images were first Gaussian-blurred with large radii (20 pixels for endophilin-A1 and 40 pixels for CAST, corresponding to 200 and 400 nm, respectively) and then subtracted from images blurred with smaller radii (2 pixels for endophilin-A1 and 4 pixels for CAST, corresponding to 20 and 40 nm, respectively). The resulting images were binarized, and components >25 pixels (0.0025 µm^2^) for endophilin-A1 and 100 pixels (0.01 µm^2^) for CAST were retained as reasonable estimates of nanoscale endophilin-A1 puncta and active zone areas, respectively. These masks were then used to quantify the nearest-neighbor distances between individual endophilin-A1 puncta and the closest active zones.

### Experimental design

Information on replicates is provided in the figure legends. Fluorescence imaging of synaptic activity using SypHy, iGluSnFR, or Syn-GCaMP involves hierarchical data structures, with multiple boutons nested within fields of view, fields within coverslips, and coverslips across independent neuronal cultures. This structure complicates the definition of independent sampling units for power estimation. Therefore, sample sizes were determined based on prior studies employing similar imaging approaches. While formal power analysis was not performed, sample sizes were chosen based on standard practices in the field and prior studies (Zhang et al., 2023; Aggarwal et al., 2023; Brockmann et al., 2020; Mori et al., 2021; Hamada et al., 2021). Reproducibility was further confirmed by consistent results obtained across independent neuronal preparations. Randomization and blinding were not implemented due to practical constraints inherent to the experimental procedures. To mitigate potential bias, data acquisition and analysis were performed using a semiautomated pipeline (SynActJ, a Fiji plugin), where possible, thereby limiting experimenter-dependent variability. Data were excluded when no detectable fluorescence increase was observed across the entire field of view during imaging, as this was considered indicative of issues with stimulation or neuronal viability.

### Statistical analysis

Data are presented as mean ± SEM, where *n* indicates the number of samples. For live-imaging analyses (Fig. 1, B and C, Fig. 2 A, Fig. 7, A and B, and Fig. 8 F), each data point represents a single field of view containing multiple active boutons (typically ≥10–15 boutons per image). Each image was acquired from a different neuron. Data were collected from at least two independent dishes. Each *n* corresponds to an independent field of view, representing an individual neuron. Fluorescence signals were quantified using automated analysis (SynActJ plugin in Fiji) to reduce experimenter bias. All experiments were replicated across three independent neuronal cultures. For Fig. 1 D; Fig. 2, B and C; and Fig. 8, B and C, analyses were conducted on a per-image basis using samples from a single dish. In each image, 10–15 boutons were randomly selected and manually measured using MetaMorph software, and their mean value was defined as *n* = 1 (one image). A total of 10–15 images were analyzed per condition, and all experiments were replicated across three independent neuronal cultures. Statistical analyses, including sample size determination and Pearson correlation coefficient calculations for the colocalization of target proteins with presynaptic markers in antibody-stained images, were conducted with reference to a previous study (Hamada et al., 2021). The sample sizes are indicated in the figure legends. Appropriate statistical tests were used with post hoc analyses when applicable, i.e., nonparametric Mann–Whitney test or one-way ANOVA analysis of variance with Bonferroni’s multiple comparison test. Analyses were performed using GraphPad Prism software (GraphPad Software www.graphpad.com). Differences were considered statistically significant when P < 0.05. *P < 0.05, **P < 0.01, ***P < 0.001, and ****P < 0.0001.

### Online supplemental material

This supplementary material includes additional figures that support the data presented in the main manuscript. Fig. S1 shows surface level of endogenous SV protein in CAST/ELKS-KD neurons and a screening analysis of co-condensation between CAST/ELKS and endocytosis-related proteins in the HEK cells. Fig. S2 shows interaction between CAST/ELKS and endophilin-A by immunoprecipitation in HEK293T cells. Fig. S3 shows the localization of a CAST mutant deficient in endophilin-A binding. Fig. S4 shows the localization of the endophilin-A1–E264A mutant, as well as the active zone size and synaptotagmin-1 localization in EndA1-KD and endophilin-A2-KD neurons. Fig. S5 shows the presynaptic localization of CHC and AP-2 in neurons with CAST/ELKS or EndA1-KD. Table S1 and Table S2 list (A) AAV sample plasmids used in this study; (B) cell line or bacterial expression plasmids used in this study; (C) antibodies used in this study.

## Ethics approval

All recombinant DNA and animal experiments in this study were performed following regulations and guidelines for the care and use of experimental animals and approved by the Institutional Committee for the Care and Use of Experimental Animals at the University of Yamanashi (protocol #A30-21). All experiments conformed to the Guidelines for the Proper Conduct of Animal Experiments of the Science Council of Japan (2006).

## Supplementary Material

Table S1 list (A) AAV sample plasmids used in this study; (B) cell line or bacterial expression plasmids used in this study; (C) antibodies used in this study.

Table S2 list (A) AAV sample plasmids used in this study; (B) cell line or bacterial expression plasmids used in this study; (C) antibodies used in this study.

SourceData F1 is the source file for Fig. 1.

SourceData F4 is the source file for Fig. 4.

SourceData F6 is the source file for Fig. 6.

SourceData F8 is the source file for Fig. 8.

SourceData F10 is the source file for Fig. 10.

SourceData FS1 is the source file for Fig. S1.

SourceData FS2 is the source file for Fig. S2.

SourceData FS3 is the source file for Fig. S3.

## Data Availability

All relevant data that support the findings of this study are available from the corresponding authors upon request.
