# Supplementary material for: CAST/ELKS–endophilin-A interaction ensures synaptic vesicle pool size
Source: J Cell Biol. 2026 Jul 22;225(9):e202508077. doi: 10.1083/jcb.202508077 (PMC13390633; doi:10.1083/jcb.202508077)
Supplement: Table S1 — list (A) AAV sample plasmids used in this study; (B) cell line or bacterial expression plasmids used in this study; (C) antibodies used in this study. [file jcb_202508077_tables1.docx]

**Supplemental table-1**

| RESOURCE | SOURCE | IDENTIFIER |
| --- | --- | --- |
| Antibodies | | |
| Anti-CAST antibody | house made (Ohtsuka et al., J. Cell. Biol., 2002) |  |
| Anti-ELKS antibody | house made (Deguchi-Tawarada et al., Gene to Cells., 2004) |  |
| Anti-alpha-Tubulin antibody | Oncogene | Cat# CP06 |
| Anti-GFP antibody | MBL | Cat# 598 |
| Anti-RFP antibody | MBL | Cat# M208-3 |
| Anti-MBL antibody | New England BioLabs | Cat# E8032S |
| Anti-Myc antibody | New England BioLabs | Cat# 011-21874 |
| Anti-Endophilin-A1 antibody | Santacruz | Cat# sc48378 |
| Anti-Endophilin-A2 antibody | Bethyl | Cat# A302-A349A |
| Anti-Synaptophysin1 antibody | Synaptic Systems | Cat# 101-001 |
| Anti-Synaptophysin1 antibody | Synaptic Systems | Cat# 101-004 |
| Anti-clathrin light chain antibody | Synaptic Systems | Cat# 113-011 |
| Anti-clathrin heavy chain antibody | Abcam | Cat# ab21679 |
| Anti-Dynamin-1 antibody | Proteintech | Cat# 18205-1-AP |
| Anti-Munc13 antibody | Synaptic Systems | Cat# 126-103 |
| Anti-Rim1 antibody | Synaptic Systems | Cat# 140-013 |
| Anti-RimBP2 | Proteintech | Cat# 15716-1-AP |
| Anti-Homer1 antibody | FRONTIER INSTITUTE | Cat# Homer1-Rb-Af1000 |
| Anti-rabbit IgG, HRP-linked Antibody | Cell Signaling Technology | Cat# 7074 |
| Anti-mouse IgG, HRP-linked Antibody | Cell Signaling Technology | Cat# 7076 |
| Goat anti-Mouse IgG (H+L) Cross-Adsorbed Secondary Antibody, Alexa Fluor™ 488 | Invitrogen | Cat# A-11001 |
| Goat anti-Mouse IgG (H+L) Cross-Adsorbed Secondary Antibody, Alexa Fluor™ 568 | Invitrogen | Cat# A-11004 |
| Goat anti-Rabbit IgG (H+L) Cross-Adsorbed Secondary Antibody, Alexa Fluor™ 488 | Invitrogen | Cat# A-11008 |
| Goat anti-Guinea Pig IgG (H+L) Highly Cross-Adsorbed Secondary Antibody, Alexa Fluor™ 633 | Invitrogen | Cat# A-21105 |
| RESOURCE | SOURCE | IDENTIFIER |
| Antibodies |  |  |
| Anti-Bassoon | Stressgen | Cat# VAM-PS003 |
| Anti-Synaptotagmin1 | Millipore | Cat# MAB5200 |
| Anti-Synaptotagmin1 (p65) antibody luminal domain | Synaptic Systems | Cat# 105 103CpH |
| Anti-Clathrin Heavy Chain (P1663) | Cell Signaling | Cat# 2410 |
| Anti-AP2M1 | Sigma-Aldrich | Cat# HPA069870 |
| Anti-Actin | Millipore | Cat# MAB1501 |
| Affinipure Donkey Anti-Rabbit IgG (H+L) | Jackson ImmunoResearch | RRID:AB_2340585 |
| Affinipure Goat Anti-Mouse IgG_1_ | Jackson ImmunoResearch | RRID:AB_2338461 |
| Affinipure Goat Anti-Mouse IgG_2a_ | Jackson ImmunoResearch | RRID:AB_2338462 |

| AAV sample plasmids used in this study | | |
| --- | --- | --- |
| pAAV-hSyn1-EYFP-MCS-WPRE | This study | N/A |
| pAAV-CaMKII-EYFP-MCS-WPRE | This study | N/A |
| pAAV-CaMKII (0.3)-MCS-WPRE | kindly gifted from Haruo Kasai | N/A |
| pAAV-CaMKII (0.3)-3xFlag-MCS-WPRE | This study | N/A |
| pAAV-CAGGS-EGFP-MCS-WPRE | This study | N/A |
| pAAV-CAGGS-Cre | This study | N/A |
| pAAV-CAGGS-Cre-mt | This study | N/A |
| pAAV-CaMKIIp-Syn-GCaMP8s | This study | N/A |
| pAAV.hSyn.iGluSnFR3.v857.SGZ | Addgene | Cat# 178330 |
| pAAV-CaMK0.3-3xFlag-CAST-WT | This study | N/A |
| pAAV-CaMK0.3-3xFlag-ELKS-WT | This study | N/A |
| pAAV-CaMK0.3-3xFlag-CAST-7RK-CRdelta | This study | N/A |
| pAAV-CaMK0.3-3xFlag-CAST-7RK-6R9KA | This study | N/A |
| pAAV-H1-shCAST-#1-mScarlet | This study | N/A |
| pAAV-H1-shCAST-#2-mScarlet | This study | N/A |
| pAAV-H1-shELKS-#1-mScarlet | This study | N/A |
| pAAV-H1-shELKS-#2-mScarlet | This study | N/A |
| pAAV-H1-shEndophilim-A1-#1-mScarlet | This study | N/A |
| pAAV-H1-shEndophilim-A1-#2-mScarlet | This study | N/A |
| pAAV-H1-shEndophilim-A1-#2-mScarlet | This study | N/A |
| pAAV-CaMK0.3-3xFlag-Endophilin-A1-SR-WT | This study | N/A |
| pAAV-CaMK0.3-3xFlag-Endophilin-A1-SR-E264A | This study | N/A |
| pAAV-CaMK0.3-Endophilin-A1-SR-WT | This study | N/A |
| pAAV-CaMK0.3-Endophilin-A1-SR-E264A | This study | N/A |



| Cell line plasmids used in this study | | |
| --- | --- | --- |
| pEGFP-C1 | Clontech |  |
| pEGFP-Synaptojyanin1 | This study | N/A |
| pEGFP-Dynamin1 | This study | N/A |
| pEGFP-Dynamin2 | This study | N/A |
| pEGFP-Dynamin3 | This study | N/A |
| pEGFP-Intersectin2 | This study | N/A |
| pEGFP-Endophilin-A1 | This study | N/A |
| pEGFP-Endophilin-A2 | This study | N/A |
| pEGFP-Endophilin-A3 | This study | N/A |
| pCAII-EGFP-AP2-alpha1 | This study | N/A |
| pEGFP-Clathrin Heavy Chain | This study | N/A |
| pEGFP-Clathrin Light Chain | This study | N/A |
| pEGFP-Amphiphysin | This study | N/A |
| pEGFP-Syndapin | This study | N/A |
| pEGFP-Rim1 | Ohtsuka et al J. Cell Biol., 2002 | N/A |
| pEGFP-Endophilin-A1-BAR | This study | N/A |
| pEGFP-Endophilin-A1-SH3 | This study | N/A |
| pEGFP-Endophilin-A2-BAR | This study | N/A |
| pEGFP-Endophilin-A2-SH3 | This study | N/A |
| pEGFP-Endophilin-A1-SR | This study | N/A |
| pEGFP-Endophilin-A1-E264A-SR | This study | N/A |
| pALFA-mCherry-C1 | This study | N/A |
| pALFA-mCherry-CAST | This study | N/A |
| pALFA-mCherry-ELKS | This study | N/A |
| pALFA-mCherry-Endophilin-A2 | This study | N/A |
| pALFA-mCherry-CAST-1-400 | This study | N/A |
| pALFA-mCherry-CAST-401-957 | This study | N/A |
| pALFA-mCherry-CAST-7RKA | This study | N/A |
| pALFA-mCherry-CAST-deltaCR | This study | N/A |
| pALFA-mCherry-CAST-7RKA-deltaCR | This study | N/A |
| pALFA-mCherry-CAST-7RKA-6R9KA | This study | N/A |
| piRFP670-C1 | This study | N/A |
| piRFP670-Endophilin-A2 | This study | N/A |
| piRFP670-Endophilin-A2-BAR | This study | N/A |
| pSUPER.neo | origene |  |
| pSUPER.neo+GFP | origene |  |
| pSUPER.neo+mScarlet | This study | N/A |
| pSUPER.neo-shCAST1392 | This study | N/A |
| pSUPER.neo-shCAST1428 | This study | N/A |
| pSUPER.neo-shELKS171 | This study | N/A |
| pSUPER.neo-shELKS468 | This study | N/A |
| pSUPER.neo+mScarlet-shEndophilin-A1-1 | This study | N/A |
| pSUPER.neo+mScarlet-shEndophilin-A1-2 | This study | N/A |
| pSUPER.neo+mScarlet-shEndophilin-A1-3 | This study | N/A |

| Bacterial expression plasmids used in this study | | |
| --- | --- | --- |
| pGEX-4T1 | Cytiva |  |
| pMAL-c2 | New England Biolabs |  |
| pET28a | Novagen |  |
| pET28a-GST | This study | N/A |
| pET28a-GST-HRV-Endophilin-A1-FL | This study | N/A |
| pET28a-GST-HRV-Endophilin-A2-FL | This study | N/A |
| pET28a-GST-HRV-Endophilin-A2-BAR | This study | N/A |
| pET28a-GST-HRV-Endophilin-A3-BAR | This study | N/A |
| pET28a-GST-myc-Dynamin1-GED-PRD | This study | N/A |
| pMAL-c2-mCAST-1-400 | This study | N/A |
| pMAL-c2-mCAST-1_7RKA-6R9KA | This study | N/A |
| pMAL-c2-mCAST-1_7RKA-deltaCR | This study | N/A |
