# Supplementary figures and images for: CAST/ELKS–endophilin-A interaction ensures synaptic vesicle pool size
Source: J Cell Biol. 2026 Jul 22;225(9):e202508077. doi: 10.1083/jcb.202508077 (PMC13390633; doi:10.1083/jcb.202508077)

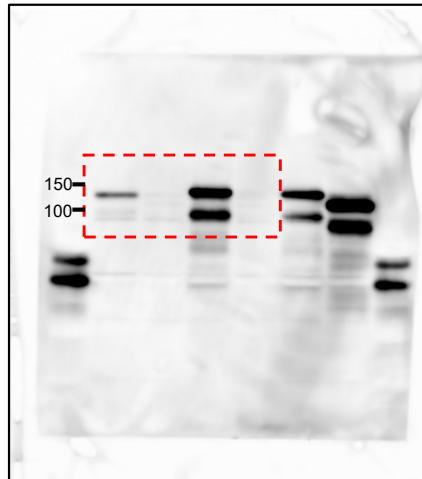

Anti-CAST

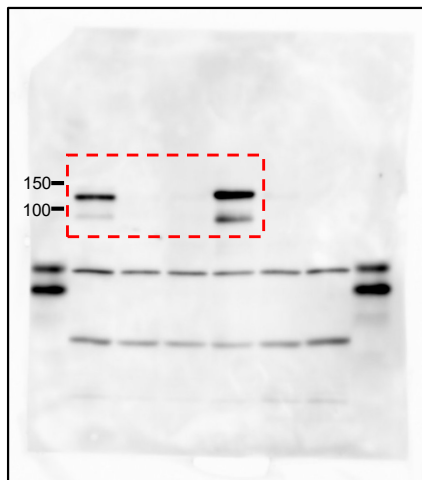

Anti-ELKS

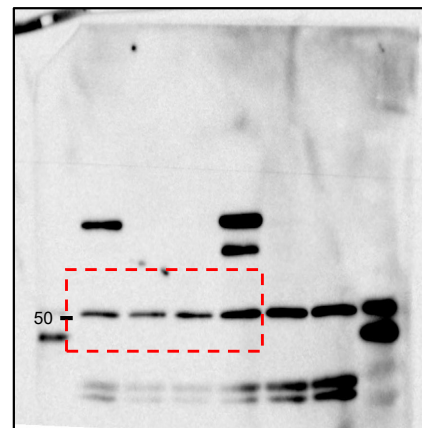

Anti-Tubulin

Supplement: SourceData F1 — is the source file for Fig. 1. [file jcb_202508077_sourcedataf1.pdf]

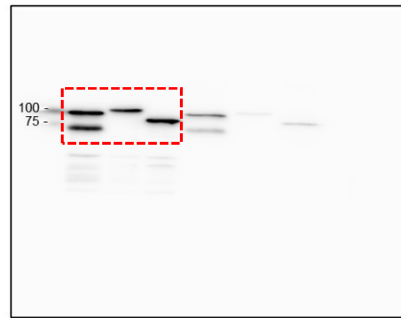

Anti-MBP  
(total lysate)

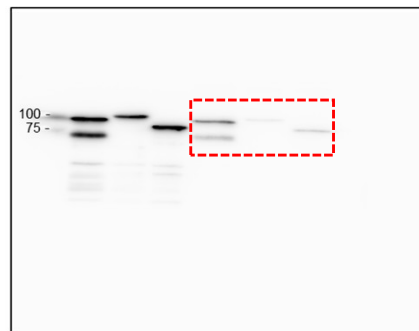

Anti-MBP  
(GST pulldown)

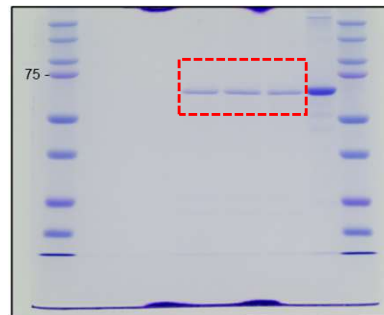

GST-Endophilin-A1-FL  
(CBB)

Supplement: SourceData F6 — is the source file for Fig. 6. [file jcb_202508077_sourcedataf6.pdf]

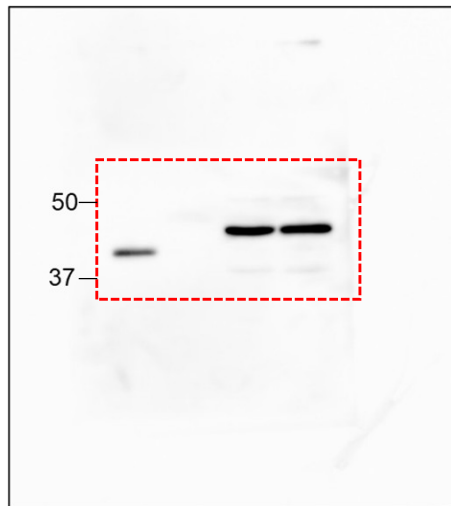

Anti-Endophilin-A1

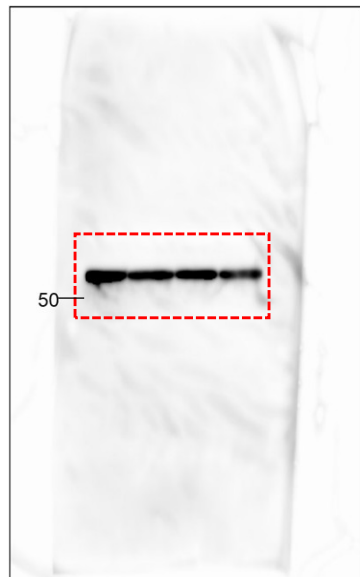

Anti-Tubulin

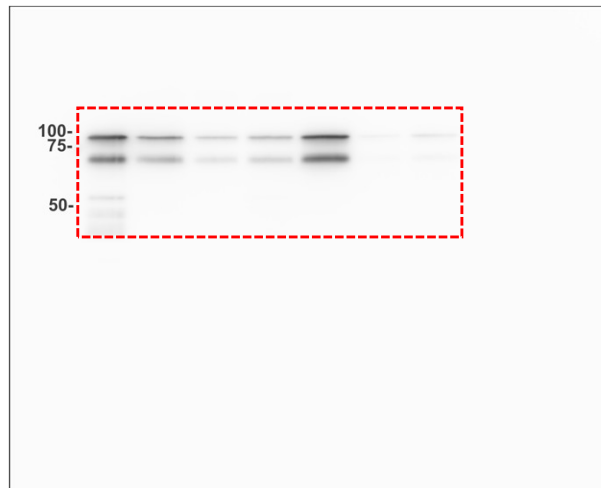

Anti-MBP  
(pull down)

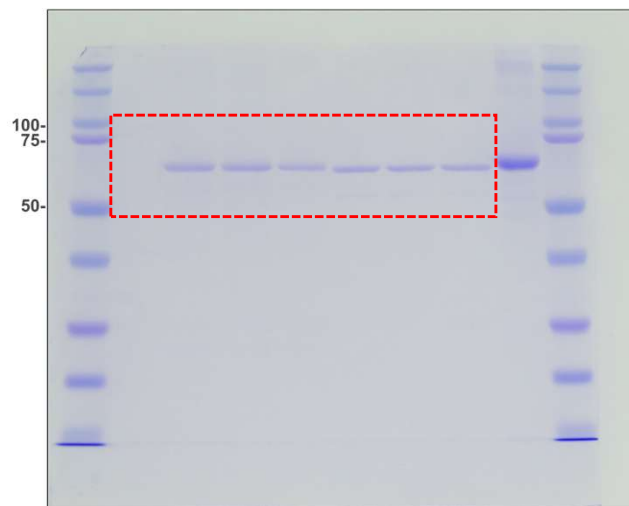

GST-Endophilin-A1  
(CBB))

Supplement: SourceData F8 — is the source file for Fig. 8. [file jcb_202508077_sourcedataf8.pdf]

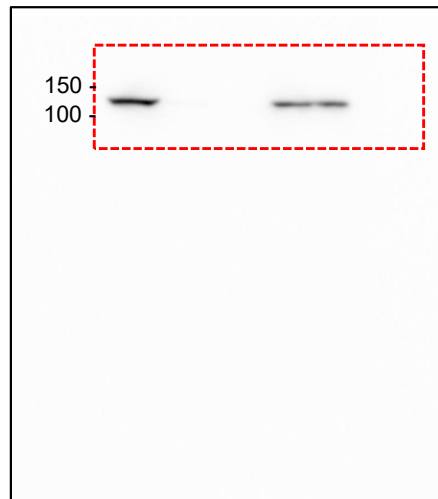

Anti-CAST

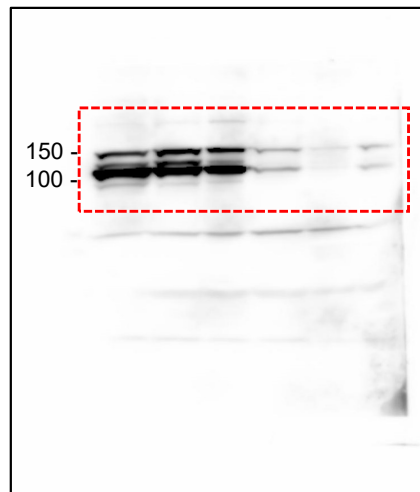

Anti-ELKS

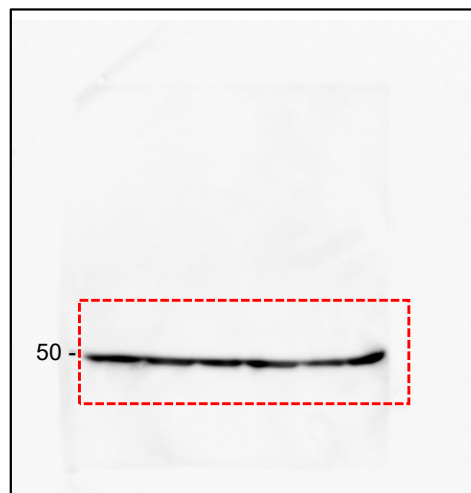

Anti-Actin

Supplement: SourceData FS1 — is the source file for Fig. S1. [file jcb_202508077_sourcedatafs1.pdf]

A

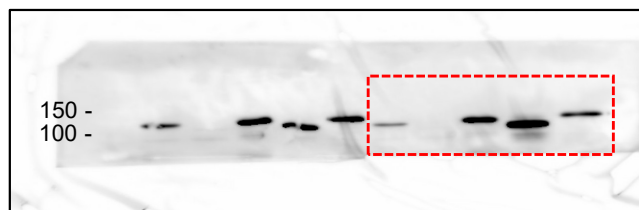

Anti-CAST

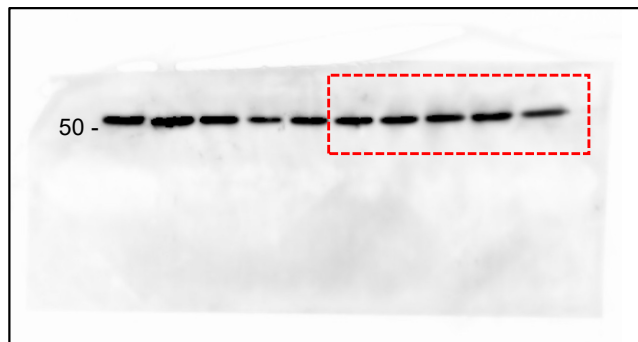

Anti-Tubulin

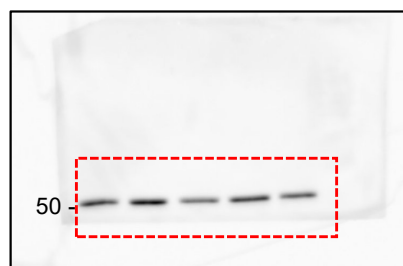

Anti-Endophilin-A1

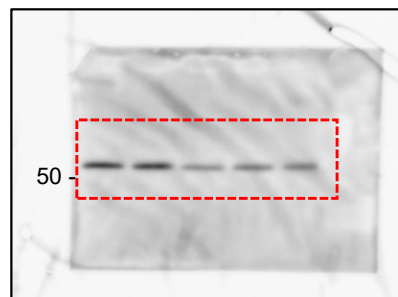

Anti-Endophilin-A2

Supplement: SourceData FS3 — is the source file for Fig. S3. [file jcb_202508077_sourcedatafs3.pdf]
