# Supplementary material for: CAST/ELKS–endophilin-A interaction ensures synaptic vesicle pool size
Source: J Cell Biol. 2026 Jul 22;225(9):e202508077. doi: 10.1083/jcb.202508077 (PMC13390633; doi:10.1083/jcb.202508077)
Supplement: SourceData F4 — is the source file for Fig. 4. [file jcb_202508077_sourcedataf4.pdf]

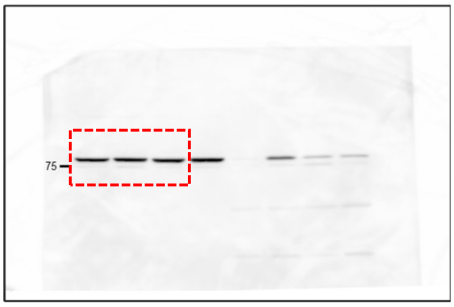

Anti-GFP
(total lysate)

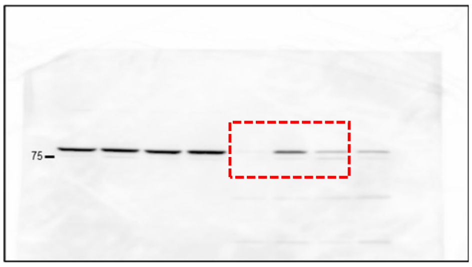

Anti-GFP
(ALFA-tag
precipitation)

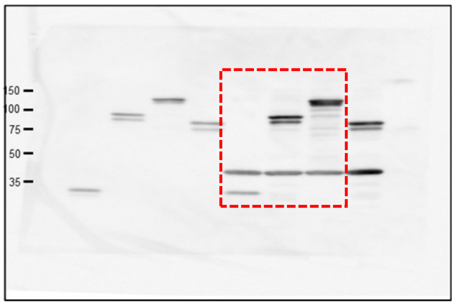

Anti-RFP
(ALFA-tag
precipitation)

Mori et al., Fig.4A

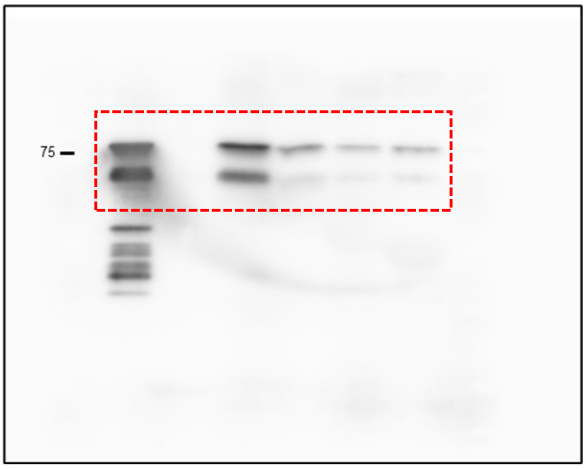

Anti-MBP
(pull down)

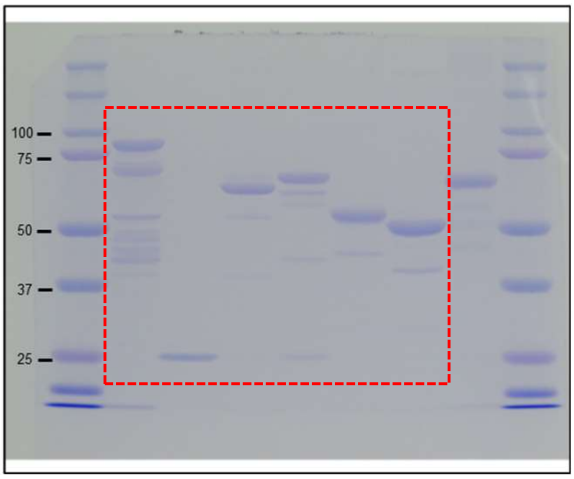

GST-tagged proteins
(CBB)

Mori et al., Fig. 4B

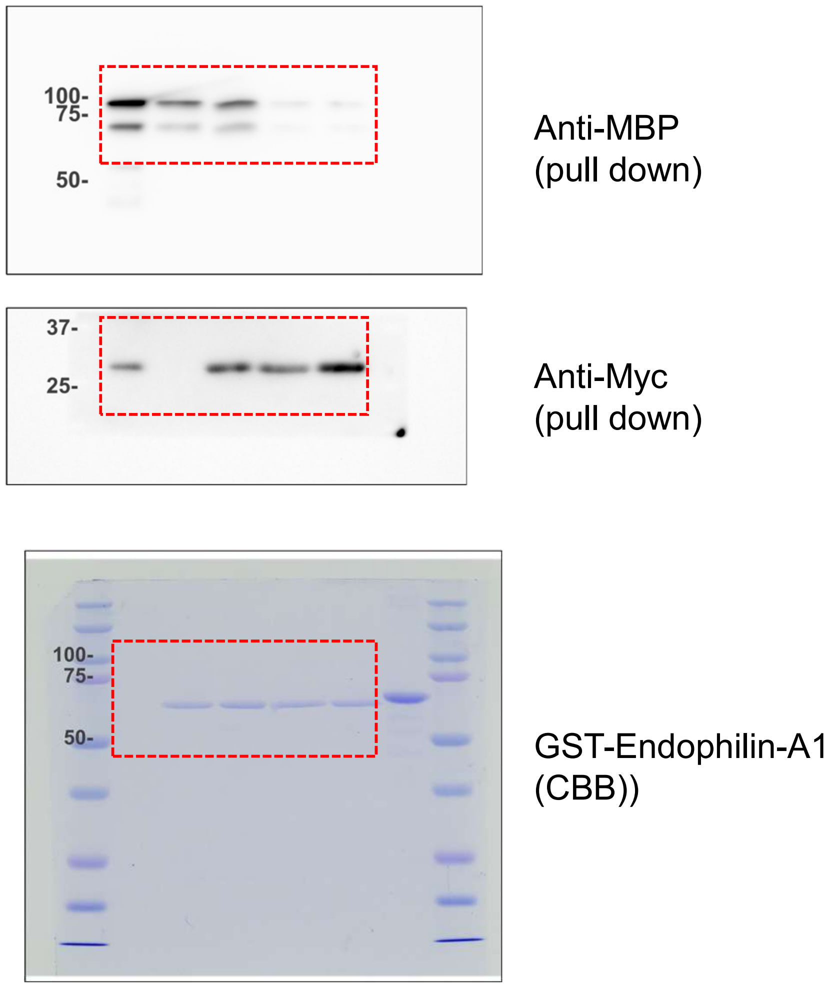

Mori et al., Fig.4C
