# Supplementary material for: CAST/ELKS–endophilin-A interaction ensures synaptic vesicle pool size
Source: J Cell Biol. 2026 Jul 22;225(9):e202508077. doi: 10.1083/jcb.202508077 (PMC13390633; doi:10.1083/jcb.202508077)
Supplement: SourceData F10 — is the source file for Fig. 10. [file jcb_202508077_sourcedataf10.pdf]

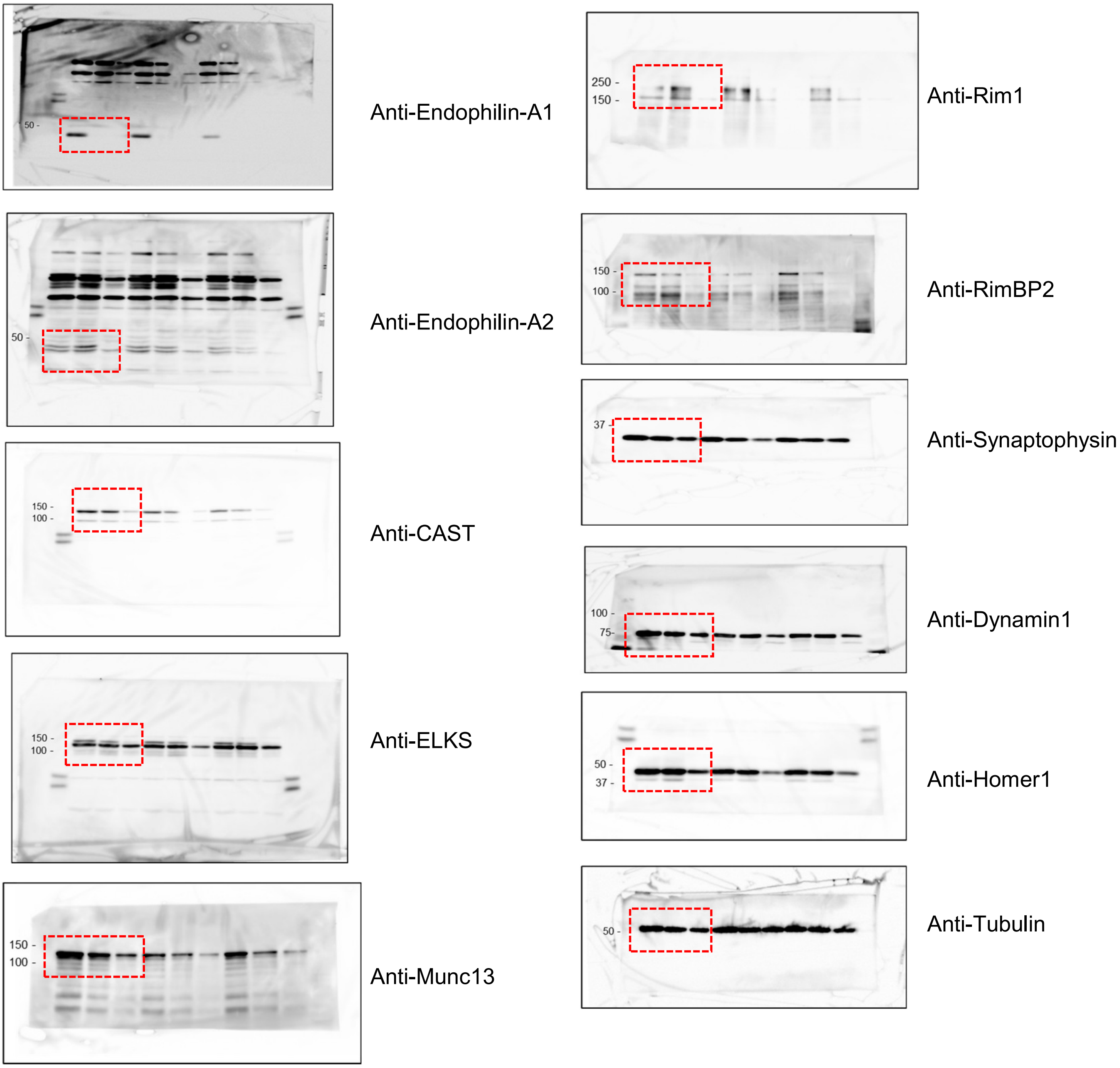

Mori et al., Fig.10A

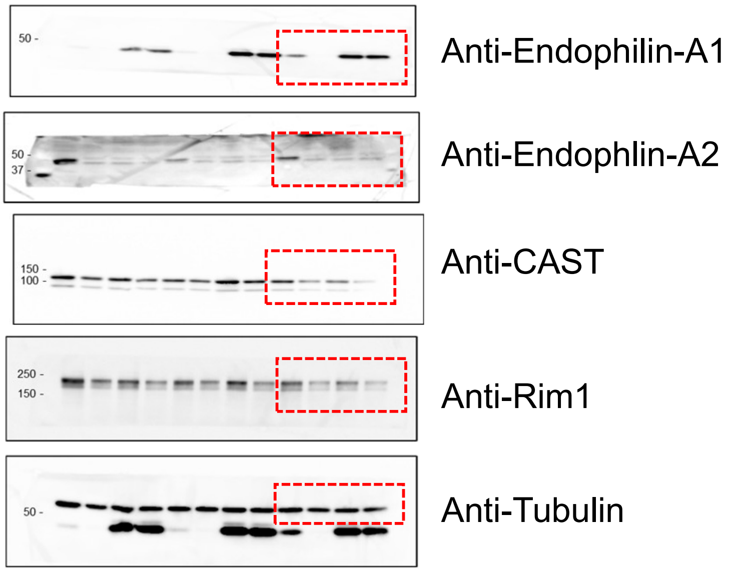

Mori et al., Fig.10C
