# Supplementary material for: CAST/ELKS–endophilin-A interaction ensures synaptic vesicle pool size
Source: J Cell Biol. 2026 Jul 22;225(9):e202508077. doi: 10.1083/jcb.202508077 (PMC13390633; doi:10.1083/jcb.202508077)
Supplement: SourceData FS2 — is the source file for Fig. S2. [file jcb_202508077_sourcedatafs2.pdf]

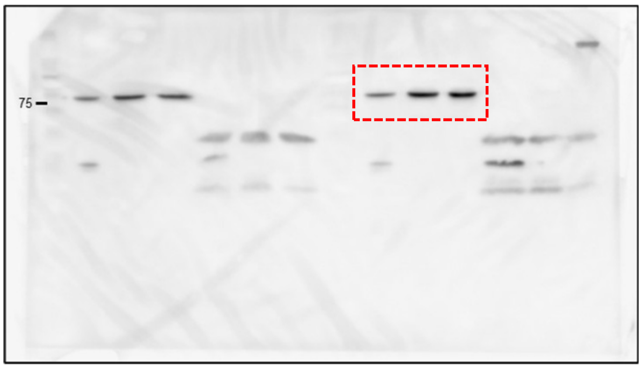

Anti-GFP (total lysate)

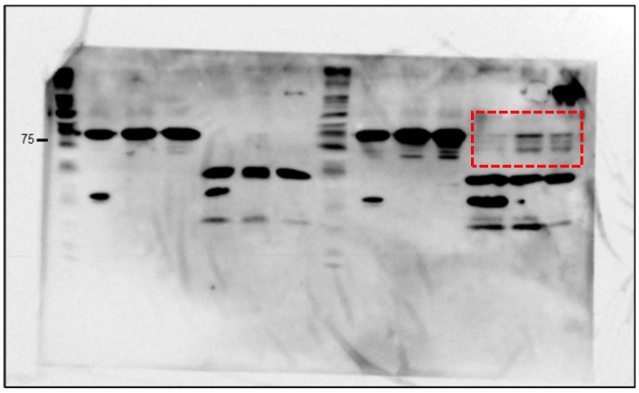

Anti-GFP (ALFA-tag precipitation)

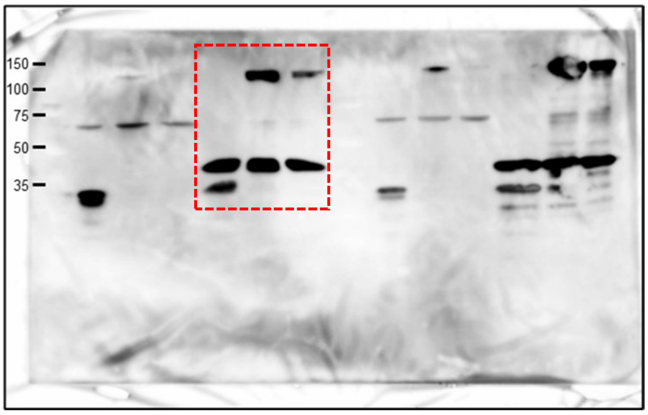

Anti-RFP (ALFA-tag precipitation)

Mori et al., Fig.S2B

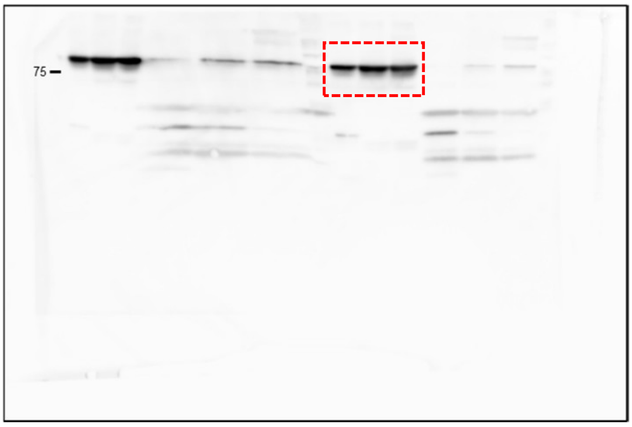

Anti-GFP (total lysate)

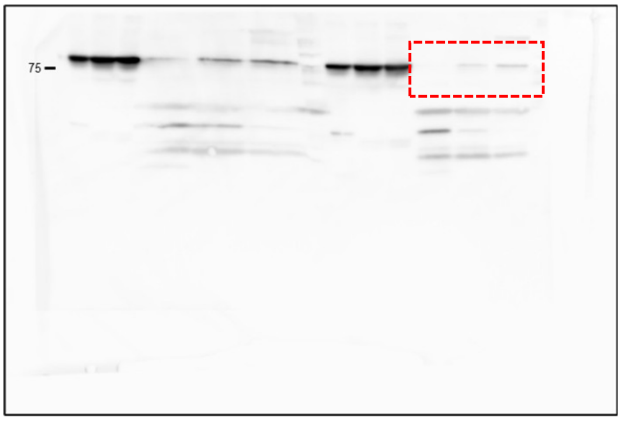

Anti-GFP (ALFA-tag precipitation)

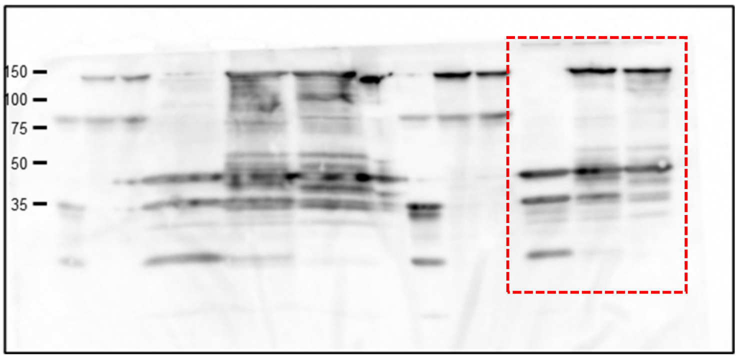

Anti-RFP (ALFA-tag precipitation)

Mori et al., Fig.S2C

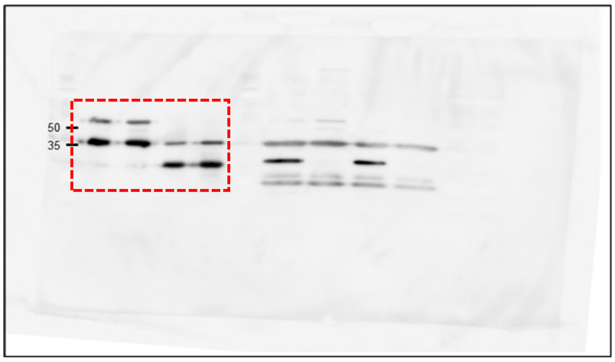

Anti-GFP (total lysate)

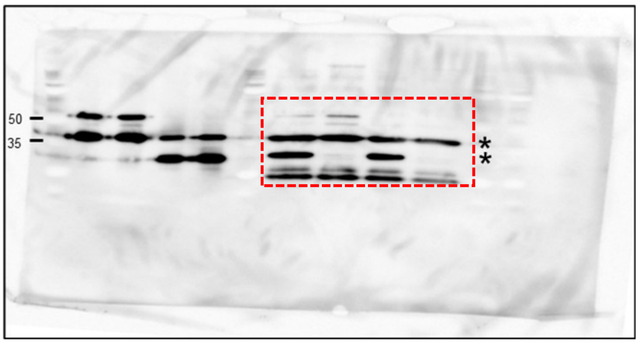

Anti-GFP (ALFA-tag precipitation)

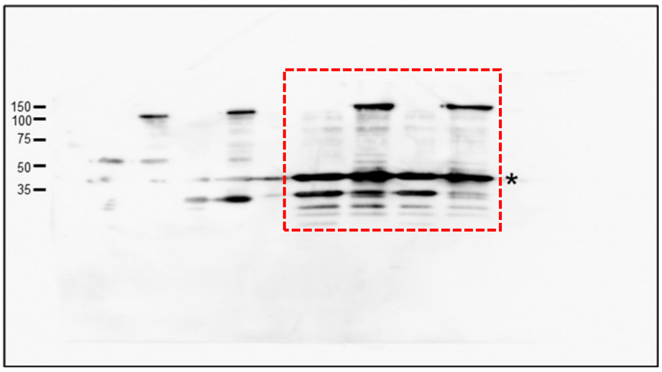

Anti-RFP (ALFA-tag precipitation)

Mori et al., Fig.S2D

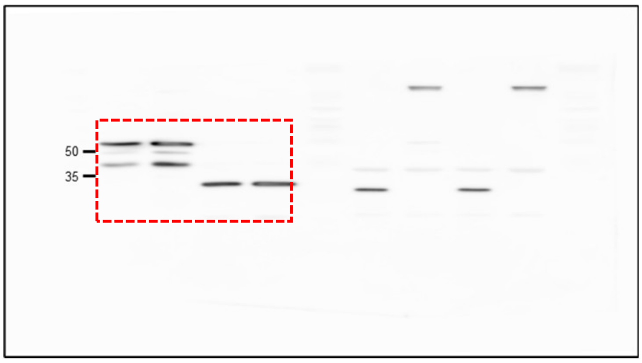

Anti-GFP (total lysate)

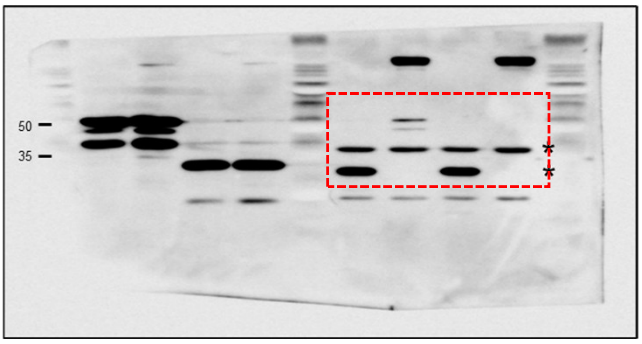

Anti-GFP (ALFA-tag precipitation)

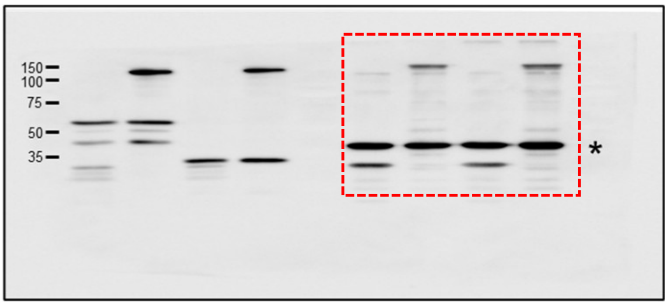

Anti-RFP (ALFA-tag precipitation)

Mori et al., Fig.S2E
